# Precise Control of Lead Halide and Ammonium Salt Stoichiometric Ratios for Efficient Perovskite Solar Cells

**DOI:** 10.1002/advs.202416634

**Published:** 2025-03-20

**Authors:** Hengyi Jiang, Rui Yang, Ziqi Zhu, Chao Sun, Yongbin Jin, Lingfang Zheng, Lina Shen, Chengbo Tian, Liqiang Xie, Jinxin Yang, Zhanhua Wei

**Affiliations:** ^1^ Xiamen Key Laboratory of Optoelectronic Materials and Advanced Manufacturing, Institute of Luminescent Materials and Information Displays, College of Materials Science and Engineering Huaqiao University Xiamen 361021 China

**Keywords:** deposition surface density, inkjet printing, organic salt, perovskite solar cells, quantitative deposition

## Abstract

The precise stoichiometric ratio of lead halide and organic ammonium salts is a fundamental yet unresolved scientific challenge in perovskite solar cells (PSCs). Conventional deposition techniques fail to establish a definitive structure‐performance relationship due to limitations in quantitative control, leading to inconsistent film quality and ambiguous reaction pathways. In this work, a precise quantitative deposition approach using drop‐on‐demand inkjet printing to systematically investigate the impact of organic salt deposition surface density on PSC performance is developed. The findings reveal that the deposition amount significantly affects the morphology, composition, and crystallinity of the perovskite films, influencing the overall device performance. Low deposition surface densities below 22 µg cm^−2^ produce thin perovskite films with incomplete crystallization and small crystals, hindering charge carrier transport and separation. Conversely, a high deposition density (89 µg cm^−2^) results in over‐reaction between the organic salt and PbI_2_, leading to low‐quality perovskite films with pinholes, cracks, and poor interfacial contact. At the optimal deposition density of 39 µg cm^−2^, it achieves high‐quality perovskite films with large grains, reduced defects, and improved energy level alignment, resulting in a champion efficiency of 23.3% and improved environmental stability for the devices.

## Graphical abstract

Herein, a quantitative deposition method is developed through drop‐on‐demand inkjet printing to study the influence of organic salt deposition surface density on the device performance. Through quantitative organic salt deposition, high‐quality perovskite films with optimal film thickness and minimized defects can be achieved. As a result, both the efficiency and stability of the devices are significantly enhanced.

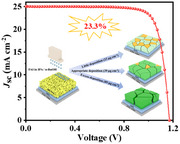

## 1 Introduction

As a cutting‐edge photoelectric material, organic–inorganic hybrid lead halide perovskites have garnered significant attention in the photovoltaic community, quickly becoming a focal point of research. Over the past decade, perovskite solar cells (PSCs) have achieved remarkable progress. During this brief period, their energy conversion efficiency has surged from an initial 3.8%^[^ ^1^ ^]^ to an impressive 26.7%.^[^ ^2^ ^]^ This rapid advancement is attributed to the myriad advantages of perovskite materials, which include tunable band gaps,^[^ ^3^, ^4^, ^5^ ^]^ exceptionally long carrier lifetimes,^[^ ^6^ ^]^ adjustable composition,^[^ ^7^, ^8^ ^]^ and high light absorption coefficients.^[^ ^9^, ^10^ ^]^ Despite this progress, the precise control over the stoichiometric ratio of lead halide and organic ammonium salts remains a critical scientific challenge, as it significantly influences the crystallinity, defect density, and interfacial properties of the resulting perovskite films.

The two‐step method has been widely applied for fabricating n‐i‐p PSCs owing to its ability to prepare perovskite films with enhanced uniformity and crystallinity.^[^ ^11^, ^12^, ^13^ ^]^ The two‐step spin coating technique is extensively employed in laboratory research due to its simplicity and high throughput screening.^[^ ^14^ ^]^ Initially, a lead halide precursor solution is spin‐coated onto a cleaned substrate and subsequently annealed to form a uniform lead halide layer. Following this, an organic halide precursor solution is spin‐coated onto the lead halide layer, and the film is subjected to a final annealing step to induce the formation of the perovskite phase, leading to the final perovskite film. The final device performance strongly depends on the quality of the perovskite film prepared by the two‐step method, which is influenced by the perovskite crystallinity,^[^ ^15^ ^]^ composition,^[^ ^16^ ^]^ residual lead iodide (PbI_2_) amount and distribution,^[^ ^17^ ^]^ and defects of the perovskite film.^[^ ^18^ ^]^

Overall, the two‐step spin coating method is particularly suitable for the rapid preparation of small‐area samples. Nevertheless, the spin coating method faces significant challenges in producing large‐area perovskite films, particularly in maintaining uniformity and consistent thickness.^[^ ^19^ ^]^ Additionally, the material utilization rate is notably low, with substantial losses occurring during spinning, limiting its broader application in commercial settings.^[^ ^20^, ^21^ ^]^ At the same time, achieving precise quantitative deposition with spin coating remains a significant challenge, complicating researchers’ ability to establish a definitive correlation between deposition parameters and device performance. In stark contrast, inkjet printing technology offers a mask‐free, non‐contact coating approach that affords exact control over material deposition and the formation of intricate patterns.^[^ ^22^, ^23^, ^24^ ^]^ Utilizing a computer‐controlled printhead, drop‐on‐demand inkjet printing can accurately deposit perovskite inks onto the substrate surface, providing quantitative ink deposition and versatility in print shape,^[^ ^25^, ^26^ ^]^ thickness,^[^ ^27^ ^]^ and film composition.^[^ ^28^ ^]^ Inkjet printing offers significant cost‐effectiveness for large‐scale PSCs manufacturing by enabling precise, drop‐on‐demand deposition, and minimizing material waste.^[^ ^29^, ^30^ ^]^ Its compatibility with roll‐to‐roll processing and existing industrial infrastructure enhances scalability, while optimized ink formulations ensure uniform film quality. The inkjet printing technique also faces challenges such as ink compatibility, nozzle clogging, and stable droplet formation.^[^ ^31^ ^]^ Incompatible inks can lead to inconsistent deposition while nozzle clogging can disrupt the printing process. Additionally, uniform droplet coalescence is crucial for consistent film quality. Thus, it's essential to optimize ink formulations by adjusting the rheological properties to ensure compatibility with the printing system. The printing parameters such as printing frequency, applied voltage, and waveform should be optimized. Although specialized equipment and tailored inks require initial investment, the long‐term benefits of reduced material costs and high‐throughput production outweigh these challenges.

Inkjet printing technology has received considerable attention in the field of PSCs.^[^ ^32^, ^33^, ^34^, ^35^, ^36^, ^37^ ^]^ Li et al. demonstrated the use of inkjet‐printed PbI_2_ ink on a mesoporous TiO_2_ layer to achieve high‐performance PSCs. A uniform ink layer was obtained by precisely controlling the droplet spacing in both the x and y directions. The mesoporous structure of the TiO_2_ layer facilitated the complete coalescence of the PbI_2_ ink, which minimized random diffusion and resulted in a uniform film. This methodology enabled the production of high‐quality perovskite films following the MAI vapor reaction. As a result, PSCs with small (0.04 cm^2^) and large (2.02 cm^2^) areas achieved champion PCEs of 18.64% and 17.74%, respectively.^[^ ^38^ ^]^ Meanwhile, researchers have also utilized the advantage of quantitative deposition from inkjet printing for high‐performance PSC fabrication, which can be achieved by controlling the deposited single droplet volume and the total droplet number.^[^ ^39^, ^40^ ^]^ Xu et al.^[^ ^28^ ^]^ introduced an advanced high‐throughput technique for the automated fabrication of perovskite films with diverse compositions and exceptional reproducibility through employing a drop‐on‐demand inkjet printing approach. This method utilizes precise control over the quantitative deposition of four distinct precursor inks (FAPbI_3_, FAPbBr_3_, MAPbI_3_, and MAPbBr_3_) by regulating the volume and quantity of ejected droplets, as well as the sequencing of precursor application. This approach enabled the rapid synthesis of 25 unique perovskite film compositions, facilitating expedited screening for optimal perovskite formulations for photovoltaic applications. Eggers et al. demonstrated the fabrication of high‐quality triple‐cation perovskite films using drop‐on‐demand inkjet printing. The film thickness could be precisely controlled by adjusting the drop spacing during printing. At an optimal thickness of 1.5 µm, the perovskite films exhibited a columnar crystal structure and enhanced charge carrier lifetime, resulting in a champion device power conversion efficiency (PCE) of 21.6%.^[^ ^27^ ^]^ Recently, Wei et al. developed a quantitative deposition technique utilizing drop‐on‐demand inkjet printing to explore the impact of 2‐adamantylamine hydrochloride (2‐ADAHCl) deposition amount on the PSC performance. By optimizing the deposition to a surface density of 2.5 µg cm^−2^, they effectively minimized perovskite surface defects and enhanced interfacial contact at the perovskite/hole transport layer (HTL) interface, and the optimized devices attained a champion PCE of 24.57%.^[^ ^41^ ^]^ These results underscore the benefits of using quantitative deposition through drop‐on‐demand inkjet printing. This technique can be further employed to explore the correlation between the amount of organic salt deposited and the properties of devices in two‐step prepared PSCs.

The amount of organic salt deposited in the two‐step method for preparing PSCs significantly influences the crystallinity of the perovskite, the amount of residual PbI_2_, the presence of trap defects, and the thickness of the perovskite film. A low organic salt deposition amount results in small perovskite grains and a significant presence of unreacted PbI_2_, leading to a thin perovskite film with incomplete light absorption. Increasing the organic salt deposition amount improves perovskite crystallinity and film thickness while reducing residual PbI_2_ content. However, excessive organic salt deposition may lead to an overly thick perovskite film, introducing more defects, grain boundaries, and structural inhomogeneities, ultimately deteriorating device performance. Therefore, precise control of organic salt deposition is essential to achieving a high‐quality perovskite film with optimized residual PbI_2_ content and film thickness, minimizing defects, and enhancing both efficiency and stability. In this study, we concentrated on the fabrication of high‐quality perovskite films by varying the deposition surface density of the organic salt solution through drop‐on‐demand inkjet printing, which can be tuned by adjusting the printed spacing between two adjacent droplets (drop spacing). We systematically analyzed the morphology, composition, and crystallinity of the perovskite films prepared with a range of organic salt surface densities from 10 to 89 µg cm^−2^. The results revealed that the organic salt deposition surface density significantly influenced the quality of the perovskite films. At surface densities below 22 µg cm^−2^, the perovskite films were not fully crystallized due to insufficient organic salt deposition, resulting in thin films with small perovskite crystals and excessive residual PbI_2_, which adversely affected device performance. Conversely, at an excessive surface density of 89 µg cm^−2^, while large perovskite grains with minimal residual PbI_2_ were observed, the films exhibited large pinholes at grain boundaries and separation at the perovskite/electron transport layer (ETL) interface. Additionally, substantial defects and significant energy level mismatches at the perovskite/HTL interface were present, leading to poor device performance. Optimal results were achieved at a surface density of 39 µg cm^−2^, where high‐quality perovskite films with large grains and appropriate residual PbI_2_ were obtained. These films demonstrated prolonged charge carrier lifetimes, reduced defects, and improved energy level alignment at the perovskite/HTL interface. Consequently, devices prepared with this optimal organic salt surface density achieved a champion PCE of 23.3%, which is the highest efficiency reported for devices fabricated using the inkjet printing method. Additionally, the PSCs retained over 89% of the initial efficiency after over 2000 h storage under 20% relative humidity in ambient air, showing excellent long‐term environmental stability.

## 2 Result and Discussion

Compared with spin coating, the inkjet printing method can achieve precise and quantitative deposition of the organic salt solution, which can be applied to investigate the relation between organic salt deposition amount and the device properties. In the process of inkjet printing, a waveform is applied to the piezoelectric actuator of the nozzle to regularly eject the ink droplets to achieve stable output.^[^ ^22^ ^]^ For quantitative and uniform deposition of the organic salt solution, stable droplet ejection from the nozzle is crucial, along with the formation of a uniform film that mitigates the “coffee ring” effect after deposition. In spin coating, isopropanol (IPA) is commonly used to dissolve the organic ammonium salts for perovskite film preparation. However, due to IPA's low boiling point and high volatility, IPA‐only inks can cause nozzle clogging during inkjet printing. In this work, a dual‐solvent system consisting of n‐butanol (n‐BuOH) and IPA was employed for printing, with the concentration of the organic salt ink fixed at 59.3 mg mL^−1^ (with the FAI concentration representing the organic salt concentration). This dual‐solvent approach effectively prevents nozzle clogging during printing. Additionally, the combination of n‐BuOH and IPA, with their differing boiling points and surface tensions, induces Marangoni flow, which helps suppress the “coffee ring” effect in the printed films.^[^ ^41^, ^42^ ^]^ At a volume ratio of 1:1, stable droplet ejection was achieved (Figure S1a, Supporting Information). The drop printability of the ink can be evaluated by the inverse (*Z*) of the Ohnesorge number (*Oh*):^[^ ^22^ ^]^

(1) $$Oh=\frac{\sqrt{We}}{Re}=\frac{\eta }{{\left(\gamma \rho a\right)}^{1/2}}$$

(2) Z=1/Oh

where *η*, *γ*, *ρ*, and *a* are the dynamic viscosity, surface tension, density of the ink, and the nozzle diameter, respectively. The calculated *Z* number is 7.06 (Table S1, Supporting Information) from Equations (1) and (2), which is among the printable range of 4 ≤ *Z* ≤ 14 from the literature.^[^ ^43^ ^]^

Meanwhile, the resulting perovskite films were uniform with a minimized “coffee ring” effect (Figure S1b, Supporting Information).

**Figure 1:**
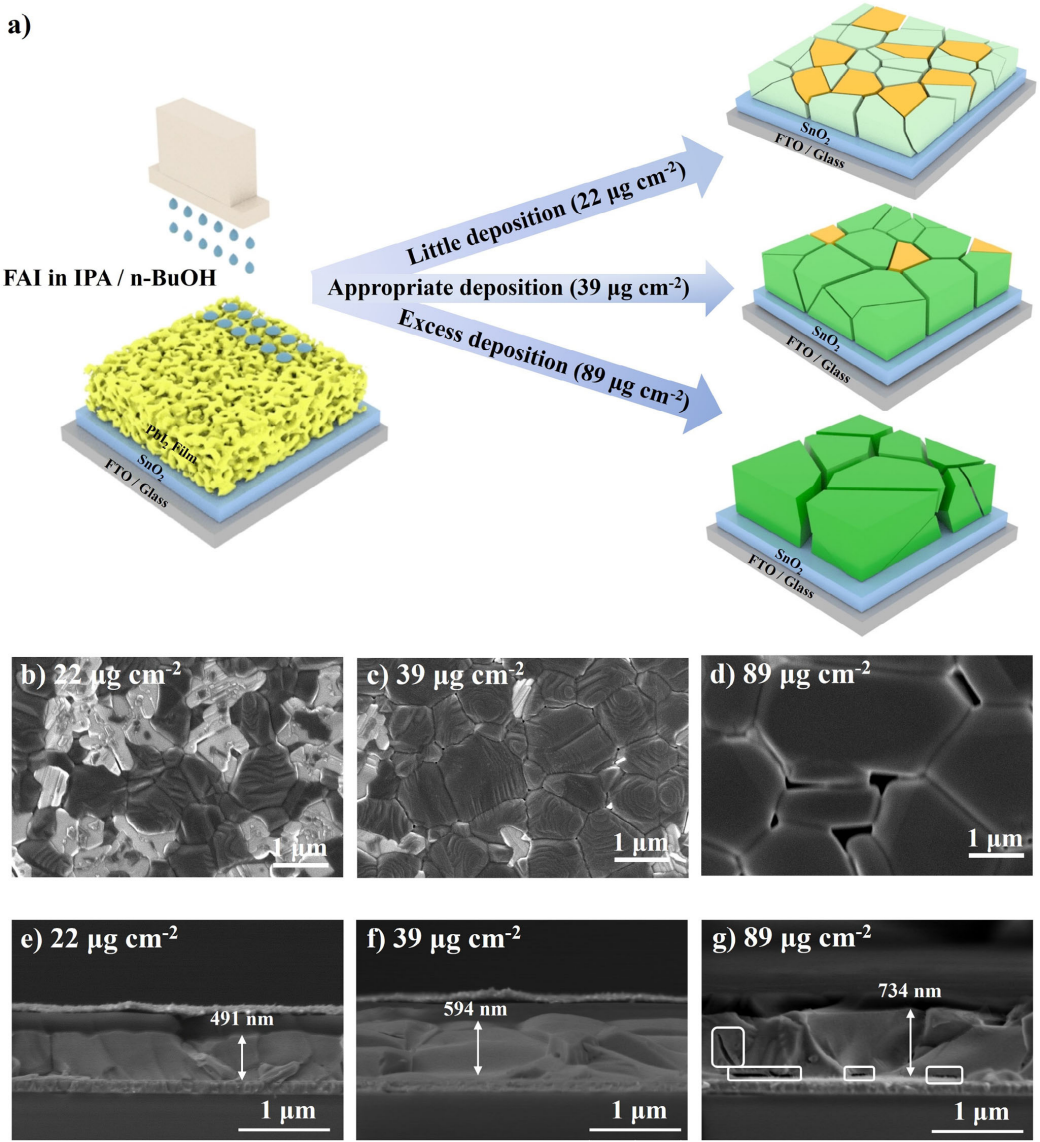
a) Schematic illustration of quantitative deposition of the organic salt solution by inkjet printing and the corresponding perovskite films at different deposition surface densities. b–d) Top‐view SEM images of the perovskite films. The perovskite grain size increased while residual PbI_2_ content decreased when increasing the deposition surface density. e–g) Cross‐sectional SEM images of the perovskite films.

The process of quantitative control of organic ammonium salt deposition by inkjet printing is briefly described in **Figure** **1**a. PbI_2_ was first spin‐coated onto the SnO_2_ layer, followed by annealing at 70 °C. Next, the organic ammonium salt ink was inkjet‐printed onto the PbI_2_ layer at different deposition amounts. Once deposited onto the PbI_2_ layer, the ink droplets, coalesced under the influence of gravity and surface tension, and ultimately merged into a liquid film. The as‐prepared perovskite film was then annealed at 150 °C for 15 min for full crystallization. The average single droplet volume from the dual‐solvent ink was calculated to be 1.5 pL. With the organic salt ink concentration fixed, the deposition surface density of organic salt is controlled by the number of droplets deposited per unit area, which is adjusted by modifying the printing drop spacing. In the subsequent experiments, drop spacings of 30, 25, 20, 15, and 10 µm were employed to regulate the surface density of organic salt deposition on the perovskite film. The corresponding surface densities were calculated as 10, 14, 22, 39, and 89 µg cm^−2^ (Table S2, Supporting Information), based on the droplet volume, ink concentration, and the number of droplets deposited per unit area.

To examine the morphological and structural changes in the perovskite films, scanning electron microscopy (SEM) was performed on films with varying organic salt deposition surface densities. As depicted in the top‐view SEM images (Figure 1b–d; Figure S2, Supporting Information), the surface morphology of the films changed significantly with different deposition surface densities. For the PbI_2_ template annealed at 70 °C, a porous structure with slight surface undulations was observed. At such a low annealing temperature, a PbI_2_‐DMSO intermediate adduct phase was formed, as reported in the literature.^[^ ^44^, ^45^ ^]^ This intermediate adduct, with its porous structure, facilitates the infiltration of organic salt solutions, promoting the formation of high‐quality perovskite films.^[^ ^46^, ^47^ ^]^ Since the final perovskite film requires annealing at 150 °C, a top‐view SEM image of the PbI_2_ layer annealed at 150 °C was also obtained for comparison. At this higher temperature, a dense film with significant surface fluctuations was observed, indicating the evaporation of DMSO and DMF, which hindered the intermolecular exchange between PbI_2_ and the organic salt, adversely affecting the formation of high‐quality perovskite films.^[^ ^45^, ^48^ ^]^ At a low organic salt deposition surface density of 10 µg cm^−2^ (Figure S2c, Supporting Information), small perovskite and PbI_2_ grains were observed. These crystals were unevenly distributed across the surface, leading to significant surface fluctuations in the perovskite film. This can be attributed to an insufficient amount of organic salt to react with the PbI_2_ template. Since drop spacing was used to adjust the deposition surface density, the distance between adjacent droplets became relatively large at lower deposition surface densities. Nucleation began once the organic salt droplets were deposited onto the PbI_2_ template. However, at such low deposition surface densities, the perovskite nuclei lacked sufficient organic salt for continued growth, leading to the formation of small perovskite grains. As the deposition surface density of the organic salt ink increased to 14, 22, and 39 µg cm^−2^, the perovskite grains grew significantly, while the residual PbI_2_ decreased substantially. It is reported that an appropriate amount of residual PbI_2_ could reduce defects and suppress nonradiative recombination of the perovskite films, while excess residual PbI_2_ could lead to hysteresis of the devices and influence long‐term stability.^[^ ^49^, ^50^ ^]^ Additionally, with the increasing deposition surface density, the perovskite and PbI_2_ grains became more aligned within the same plane. Further increasing the organic salt deposition amount to 89 µg cm^−2^ resulted in larger perovskite grains and even lower residual PbI_2_. The perovskite grain size increased with higher organic salt deposition amounts (Figure S3, Supporting Information), suggesting that enhanced organic salt deposition promoted perovskite crystallinity. This grain growth may be attributed to the Ostwald ripening process occurring at high deposition surface densities. However, large pinholes were observed at the grain boundaries of the perovskite crystals at the organic salt deposition amount of 89 µg cm^−2^, which could severely deteriorate the efficiency and long‐term stability of the PSCs.^[^ ^51^, ^52^ ^]^

To further examine the structure and thickness of the perovskite films, cross‐sectional SEM images of the PSCs were obtained, as shown in Figure 1e–g and Figure S4 (Supporting Information). At an annealing temperature of 70 °C, a relatively flat and porous PbI_2_ structure was observed, which aligns well with the top‐view SEM results. After depositing the organic salt ink at various surface densities and annealing at 150 °C, perovskite films with different morphologies were formed. At organic salt deposition surface densities of 10 and 14 µg cm^−2^, the films were primarily composed of PbI_2_ and exhibited significant non‐uniformity. As the surface density increased to 22 µg cm^−2^, columnar perovskite grains began to form. However, large PbI_2_ clusters were still present. At a surface density of 39 µg cm^−2^, a uniform perovskite film with large columnar grains was formed, and this perovskite film exhibited compact contact with both the ETL and HTL layers. With a further increase in surface density to 89 µg cm^−2^, large cracks were observed across the perovskite film and at the perovskite/ETL interface. This was possibly due to the over‐reaction between the organic salt solution and PbI_2_ at an excess organic salt deposition surface density, which resulted in cracks through the perovskite film and separation at the perovskite/ETL interface. These cracks indicated that an excessive amount of organic salt can significantly degrade the quality of the perovskite films, adversely affecting charge carrier transport in the PSCs.

The average thickness of the perovskite layers at various organic salt deposition surface densities, along with the thickness of the PbI_2_ layer annealed at 70 °C, is shown in Figure S5 (Supporting Information). The average perovskite film thickness increased from 392 to 734 nm as the organic salt deposition surface density increased from 10 to 89 µg cm^−2^. Interestingly, a decrease in film thickness was observed between the PbI_2_ layer at 70 °C and the perovskite film at a deposition surface density of 10 µg cm^−2^. To further investigate this thickness reduction, the cross‐sectional SEM image for the PbI_2_ layer annealed at 150 °C was carried out (Figure S4b, Supporting Information). Unlike the porous structure of the PbI_2_ layer annealed at 70 °C, the layer annealed at 150 °C displayed a dense structure with significant thickness variation (ranging from 212 to 505 nm), consistent with the rough surface from the top‐view SEM image. As the film at the deposition surface density of 10 µg cm^−2^ was primarily composed of PbI_2_, we concluded that the evaporation of DMF and DMSO, combined with the formation of a dense perovskite and PbI_2_ structure during annealing at 150 °C, contributed to the reduction in film thickness at a lower organic salt deposition surface density.

Atomic force microscopy (AFM) measurements were conducted to further elucidate the relationship between the organic salt deposition surface density and the surface roughness of the perovskite film, as shown in Figure S6 (Supporting Information), with the roughness values summarized in Table S3 (Supporting Information). The PbI_2_ layer annealed at 70 °C exhibited a relatively smooth surface with a roughness of 11.3 nm, while the roughness of the PbI_2_ layer annealed at 150 °C increased extensively to 70.3 nm, consistent with the SEM results. At a low organic salt deposition surface density of 10 µg cm^−2^, the perovskite film showed an increased surface roughness of 36.7 nm compared to the PbI_2_ layer annealed at 70 °C, which was attributed to the formation of small perovskite and PbI_2_ grains. As the deposition surface density increased to 14 and 22 µg cm^−2^, the roughness of the perovskite films decreased substantially to 31.8 and 14.8 nm, respectively. At the surface density of 39 µg cm^−2^, the perovskite film exhibited the lowest roughness of 12.8 nm. Moreover, further increasing the organic salt deposition amount to 89 µg cm^−2^ led to a significant rise in surface roughness (40.2 nm), which was due to the formation of large pinholes at the perovskite grain boundaries. We summarized the structure and composition of perovskite films at various organic salt deposition surface densities in Figure 1a. A low organic salt deposition surface density (below 22 µg cm^−2^) resulted in the formation of a thin perovskite film with small grains and substantial residual PbI_2_. An optimal deposition surface density (39 µg cm^−2^) produced a perovskite film with large perovskite grains, moderate residual PbI_2_, and appropriate film thickness. In contrast, an excess deposition surface density (89 µg cm^−2^) led to an overly thick perovskite film with large perovskite grains, low residual PbI_2_, but also large pinholes at the grain boundaries and cracks through the perovskite.

**Figure 2:**
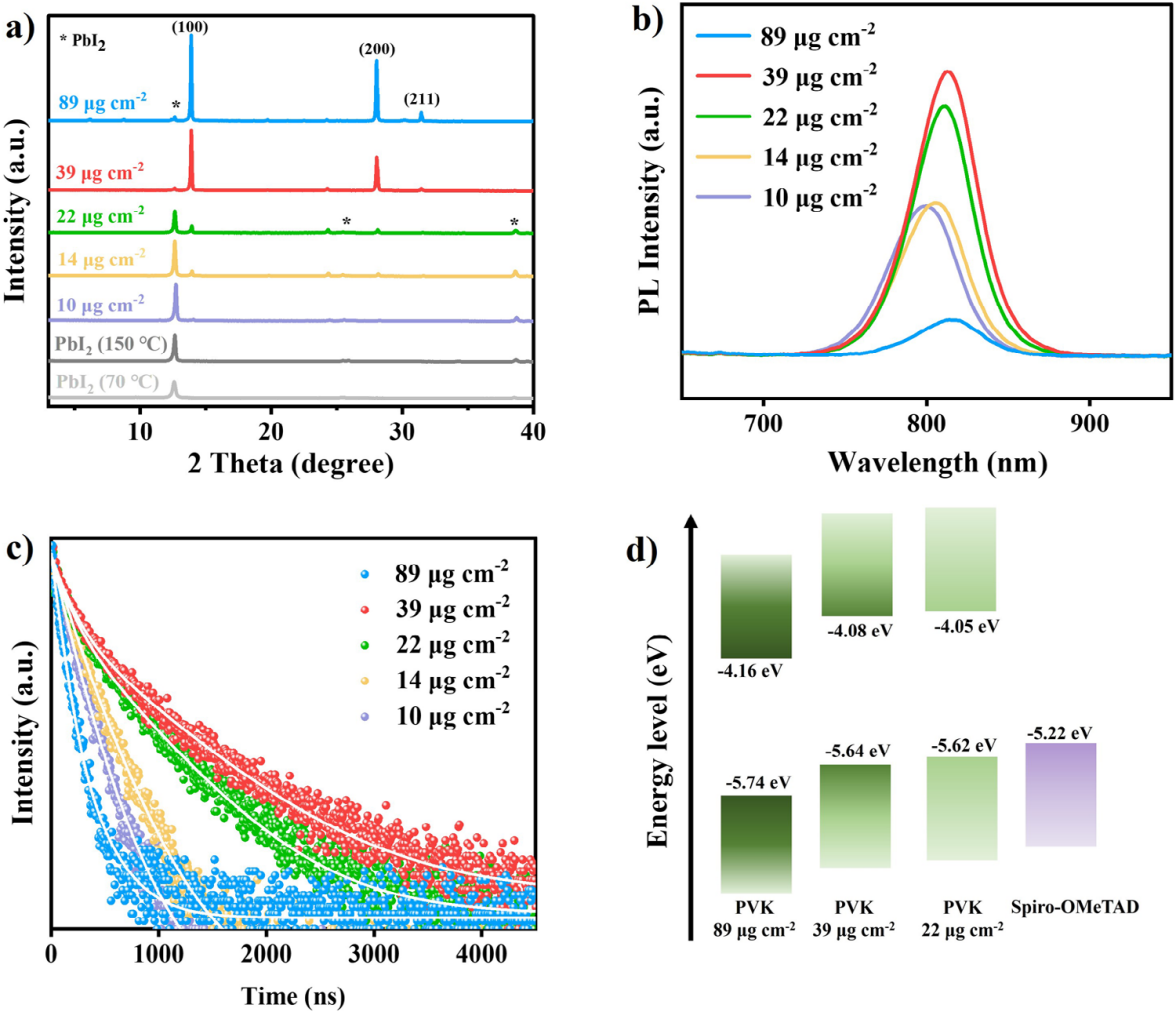
a) XRD patterns of the perovskite films at various organic salt deposition surface densities. b) Steady‐state PL analysis of the perovskite films on glass substrates. c) Time‐resolved PL decay curves of the perovskite films on glass substrates. d) Schematic illustration of the energy‐level alignment of the perovskite films at different organic salt deposition surface densities along with Spiro‐OMeTAD. PVK represents perovskite.

X‐ray diffraction (XRD) measurements were performed to examine the crystallinity and phase variations of the perovskite films at different organic salt deposition surface densities. As depicted in **Figure** **2**a, the peak located at 12.70° corresponds to PbI_2_, with significantly higher intensity for the PbI_2_ layer annealed at 150 °C compared to 70 °C. This confirms the formation of a PbI_2_‐DMSO adduct and incomplete crystallization of PbI_2_ at 70 °C. At a deposition surface density of 10 µg cm^−2^, a small peak at 14.04°, corresponding to the 𝛼‐perovskite (100) plane, was observed, with a high PbI_2_/𝛼‐perovskite (100) peak intensity ratio of 13.8 (Table S4, Supporting Information). This further confirms that PbI_2_ is the dominant component at low deposition densities. As the surface density increased from 10 to 22 µg cm^−2^, the 𝛼‐perovskite peak intensity gradually increased, and the peak intensity ratio between PbI_2_ and 𝛼‐perovskite (100) plane decreased significantly. At deposition surface densities of 39 and 89 µg cm^−2^, the 𝛼‐perovskite peaks were significantly enhanced, while the PbI_2_ peaks dramatically diminished, indicating a marked improvement in the crystallinity of the perovskite films at these higher deposition densities. Meanwhile, the perovskite film at 89 µg cm^−2^ exhibited an enlarged peak at 31.4°, corresponding to the (211) plane of 𝛼‐perovskite.^[^ ^53^ ^]^ This suggested that the orientation of the perovskite was not uniform at this high deposition surface density, which could hinder charge carrier transport.^[^ ^54^ ^]^ Additionally, two small peaks at 6.5° and 8.8° were observed in the perovskite film at 89 µg cm^−2^. This was possibly due to the presence of guanidinium ions in the formulation, which may lead to the formation of 2D alternating cation (ACI) perovskites at a high organic salt deposition surface density.^[^ ^55^, ^56^ ^]^

Steady‐state photoluminescence (PL) measurements were conducted to assess the quality of perovskite films at varying organic salt deposition surface densities, as shown in Figure 2b. A slight increase in PL peak intensity was observed as the surface density increased from 10 to 14 µg cm^−2^. The highest PL intensity was observed at 39 µg cm^−2^, followed by the film treated with 22 µg cm^−2^ of organic salt. However, further increasing the deposition surface density to 89 µg cm^−2^ led to a significant decrease in PL intensity, reaching the lowest value among the five tested gradients. Based on the above analyses, at low organic salt deposition surface densities (10, 14, and 22 µg cm^−2^), the perovskite films were thin, contained excess residual PbI_2_, and were not fully crystallized, resulting in lower PL intensities. In contrast, at a high deposition surface density of 89 µg cm^−2^, significant increases in defect density, grain boundaries, and pinholes occurred, promoting substantial non‐radiative recombination. Meanwhile, the presence of ultra‐large perovskite grains with cracks, along with non‐uniform crystal orientation, limited carrier transport in the thick perovskite film. These factors collectively led to a noticeable reduction in PL intensity. The optimal deposition surface density of 39 µg cm^−2^ produced a high‐quality perovskite film with enhanced crystallinity and fewer defects, resulting in the highest PL peak intensity. Additionally, a red shift in the PL peaks was observed with increasing organic salt deposition surface density, possibly attributed to the significant increase in film thickness at higher deposition surface densities.^[^ ^57^, ^58^ ^]^ PL mapping results (Figure S7, Supporting Information) further confirmed that a uniform and high‐quality perovskite film was achieved at a deposition surface density of 39 µg cm^−2^. In contrast, films with deposition surface densities of 10, 14, and 22 µg cm^−2^ exhibited non‐uniformity with visible stripe patterns and reduced photoluminescence. This was due to insufficient organic salt deposition onto the PbI_2_ template, which impeded the formation of a uniform perovskite film. At an excessive deposition surface density of 89 µg cm^−2^, a non‐uniform perovskite film with significantly lower photoluminescence was observed, consistent with the previous PL results.

Time‐resolved photoluminescence (TRPL) spectra were employed to investigate the charge recombination behavior of perovskite films at various organic salt deposition surface densities, as shown in Figure 2c. The decay curves were fitted with a bi‐exponential function, and the corresponding parameters are provided in Table S5 (Supporting Information). An improvement in charge carrier lifetime was observed as the deposition surface density increased from 10 µg cm^−2^ (τ_ave_ = 225 ns) to 39 µg cm^−2^ (τ_ave_ = 729 ns), while a significant reduction in carrier lifetime was recorded at an excess deposition surface density of 89 µg cm^−2^ (τ_ave_ = 147 ns). These findings further confirm that a high‐quality perovskite film with markedly suppressed non‐radiative recombination was achieved at the optimal organic salt deposition surface density of 39 µg cm^−2^. UV–vis absorption measurements were also conducted to assess the absorption behavior of the perovskite films across the various deposition surface densities (Figure S8a, Supporting Information). The absorption spectra revealed that increasing the organic salt deposition surface density resulted in enhanced absorption, which can be attributed to the formation of thicker films with larger perovskite grain sizes as the deposition surface density increased. Optical bandgap (Eg) values, calculated from Tauc plots (Figure S8b, Supporting Information), showed a decrease from 1.580 eV at 10 µg cm^−2^ to 1.545 eV at 89 µg cm^−2^. This reduction in Eg was possibly attributed to the dramatic increase in film thickness when increasing deposition surface density.^[^ ^57^ ^]^

To investigate the energy level of the perovskite films, UV photoelectron spectroscopy (UPS) was performed for films with organic salt deposition surface densities of 22, 39, and 89 µg cm^−2^ (Figure S9, Supporting Information), with the corresponding energy‐level diagrams shown in Figure 2d. A slight downshift of the valence band maximum (VBM) was observed as the deposition surface density increased from 22 µg cm^−2^ (−5.62 eV) to 39 µg cm^−2^ (−5.64 eV), while a more pronounced shift to −5.74 eV occurred for the film with at 89 µg cm^−2^. The closer alignment of the VBM to the Spiro‐OMeTAD HOMO energy level at low and moderate organic salt deposition densities suggested reduced energetic mismatch at the perovskite/HTL interface. In contrast, the substantial VBM downshift at the highest deposition density indicated a significant energetic mismatch, which could impair charge carrier transport and adversely affect the device's efficiency.^[^ ^59^, ^60^ ^]^ Based on the film characterizations, we conclude that organic salt deposition surface densities below 22 µg cm^−2^ yields thin, low‐quality perovskite films with incomplete crystallization and excess PbI_2_. Conversely, a deposition surface density of 89 µg cm^−2^ results in poor‐quality films with non‐uniform perovskite orientation, large pinholes at grain boundaries, interfacial separations at the perovskite/ETL interface, and severe energetic mismatch at the perovskite/HTL interface. At an optimal deposition surface density of 39 µg cm^−2^, high‐quality perovskite films are obtained, featuring large grains, appropriate residual PbI_2_ for defects passivation, and improved energy level alignment at the perovskite/HTL interface.

**Figure 3:**
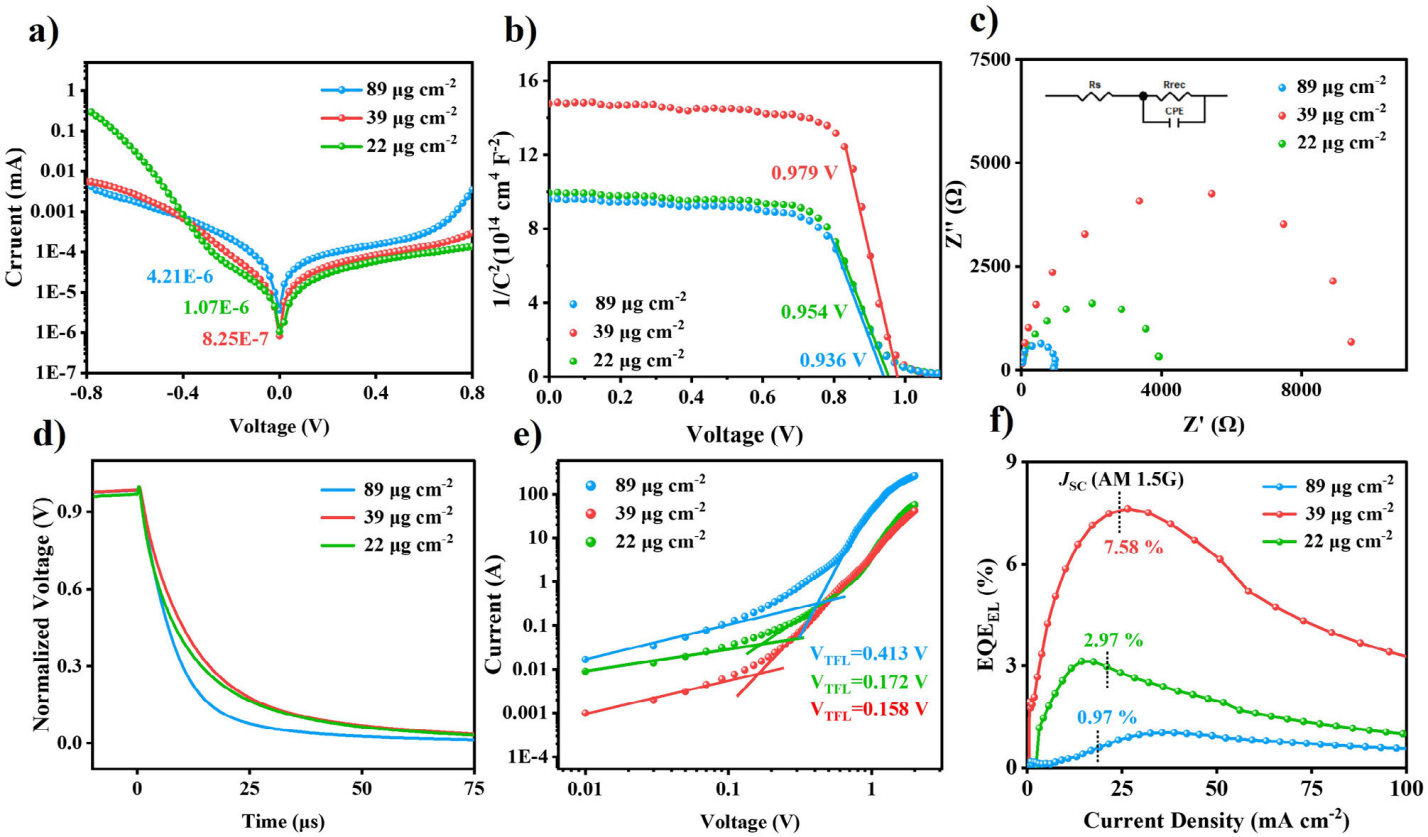
Characterization of the devices at different organic salt deposition surface densities. a) *J*–*V* curves of the devices in the dark condition. b) Mott–Schottky analysis at 1 kHz. c) Nyquist plots of the electrochemical impedance spectroscopy of the devices at a bias of 1.0 V under dark conditions. The inset showed the equivalent circuit. d) Transient photovoltage decay curves. e) SCLC results of the hole‐only devices. f) EQE_EL_ as a function of current density at electroluminescence.

To study the performance of devices at various organic salt deposition surface densities, we conducted a series of device characterizations. Based on the previous perovskite film characterization results, three groups of devices with deposition surface densities of 22, 39, and 89 µg cm^−2^ were tested. **Figure** **3**a illustrates the dark current analysis of devices prepared with these different organic salt deposition surface densities. A notable reduction in dark current density at 0 V was observed for the device prepared with 39 µg cm^−2^ organic salts compared to that with 89 µg cm^−2^, indicating effective suppression of leakage current.^[^ ^61^ ^]^ The Mott–Schottky analysis (Figure 3b) revealed varying built‐in potential (V_bi_) values for each group. The V_bi_ values were found to be 0.954, 0.979, and 0.936 V for devices prepared with 22, 39, and 89 µg cm^−2^ of organic salt, respectively. The higher V_bi_ value in the 39 µg cm^−2^ organic salt‐prepared devices indicated enhanced photogenerated carrier separation and transport, which could contribute to an improved open‐circuit voltage (*V*_OC_).^[^ ^62^ ^]^ Figure 3c presents the Nyquist plots derived from the electrochemical impedance spectroscopy (EIS) tests. The charge recombination resistance (R_rec_), extracted using the inset equivalent circuit, was measured to be 3533, 8858, and 1190 Ω for devices prepared with 22, 39, and 89 µg cm^−2^ of organic salt, respectively. These results indicated that the device prepared with 39 µg cm^−2^ of organic salt demonstrated the highest carrier separation capability, while the device with 89 µg cm^−2^ of organic salt showed the lowest. To further examine the carrier recombination dynamics, transient photovoltage measurements were conducted on devices with varying organic salt deposition surface densities. As shown in Figure 3d, the device prepared with 39 µg cm^−2^ of organic salt exhibited the longest *V*_OC_ decay lifetime, indicating superior suppression of recombination and enhanced charge separation. To evaluate the defect states of the perovskite films, space charge limited current (SCLC) measurements were performed through the hole‐only devices with the structure of FTO/PEDOT: PSS/perovskite/Spiro‐OMeTAD/Ag. As shown in Figure 3e, the trap‐filled limit voltage (*V*_TFL_) was significantly lower for the devices prepared with 39 µg cm^−2^ of organic salt (0.158 V) compared to those with other deposition surface densities (0.172 V for 22 µg cm^−2^ and 0.413 V for 89 µg cm^−2^). The trap state density of the perovskite films can then be evaluated by *V*_TFL_ and the following equation:

(3) $$n_{\mathrm{trap}}=\frac{\varepsilon_{0}\varepsilon_{r}V_{\mathrm{TFL}}}{\;eL^{2}}$$

where *ε_0_*, *ε_r_*, *e*, and *L* are the vacuum permittivity, relative permittivity of the material, elementary charge, and the thickness of the perovskite film, respectively. The trap state densities were calculated to be 2.05 × 10^15^, 1.29 × 10^15^, and 2.21 × 10^15^ cm^−3^ for the films prepared with 22, 39, and 89 µg cm^−2^ organic salts, respectively. To investigate the surface Pb/I ratio and defects of the perovskite films, X‐ray photoelectron spectroscopy (XPS) was performed at various organic salt deposition surface densities (Figures S10 and S11, Supporting Information). The Pb: I elemental ratios for the perovskite films were 1:2.85, 1:3.03, and 1:3.10 at surface densities of 22, 39, and 89 µg cm⁻^2^, respectively. This trend indicated that as the organic salt deposition surface density increased, the film surface composition approached the ideal Pb/I ratio of 1:3 of the perovskite structure. The small peaks in the Pb XPS spectra were identified as metallic Pb (Pb^0^) peaks.^[^ ^63^ ^]^ The integral area ratio of Pb^0^/(Pb^0^ + Pb^2+^) decreased from 6.09% at 22 µg cm^−2^ to 5.85% and 5.83% at 39 and 89 µg cm^−2^, respectively. This suggested that Pb^0^ defects were significantly suppressed when the deposition surface density increased from 22 to 39 µg cm^−2^, but a further increase in the surface density to 89 µg cm^−2^ had little additional effect on the suppression of Pb^0^ defects. Additionally, the I *3d* peaks shifted to lower binding energy values with increasing deposition surface densities, indicating a possible reduction in iodine vacancies.^[^ ^64^ ^]^ The Pb *4f* peaks, on the other hand, shifted to higher binding energies, suggesting an increase in defects associated with undercoordinated Pb^2+^ as the deposition surface density increased.^[^ ^65^ ^]^ Overall, the analysis revealed that different types of defects dominated the perovskite surface at different organic salt deposition surface densities, with the perovskite film at 39 µg cm^−2^ showing the lowest trap state density.

To further assess non‐radiative recombination in the devices, external quantum efficiency (EQE) of electroluminescence (EQE_EL_) tests were conducted for devices with different organic salt deposition surface densities. As shown in Figure 3f, the device prepared with 39 µg cm^−2^ of organic salt yielded the highest EQE_EL_ value (7.58%) at an injection current density equal to the short‐circuit current density (*J*_SC_) under 1 sun illumination (Table S6, Supporting Information), significantly outperforming devices prepared with 22 µg cm^−2^ (2.97%) and 89 µg cm^−2^ (0.97%) of organic salt. The *V*_OC_ loss of PSCs can be calculated using the following equation:

(4) $$\Delta V_{\mathrm{OC}}=\Delta V_{\mathrm{OC},\mathrm{rad}}+\Delta V_{\mathrm{OC},\mathrm{nonrad}}=\Delta V_{\mathrm{OC},\mathrm{rad}}+\frac{k_{B}T}{q}\ln{\mathrm{EQE}}_{\mathrm{EL}}$$

Using the measured EQE_EL_ values and the corresponding equation, the non‐radiative recombination induced Δ*V*_OC_ was estimated to be only 66.79 mV for the device prepared with 39 µg cm^−2^ of organic salt. In contrast, devices with insufficient or excess organic salt deposition surface densities exhibited significantly higher Δ*V*_OC_ values, at 91.04 mV (22 µg cm^−2^) and 120.20 mV (89 µg cm^−2^), due to increased non‐radiative recombination. These findings suggested that trap‐assisted recombination was substantially minimized at the optimal organic salt deposition surface density of 39 µg cm^−2^, contributing to a significant improvement in *V*_OC_. Conversely, devices with insufficient or excess organic salt deposition suffered from more defect‐assisted recombination, correlating with the poor quality of the perovskite films.

**Figure 4:**
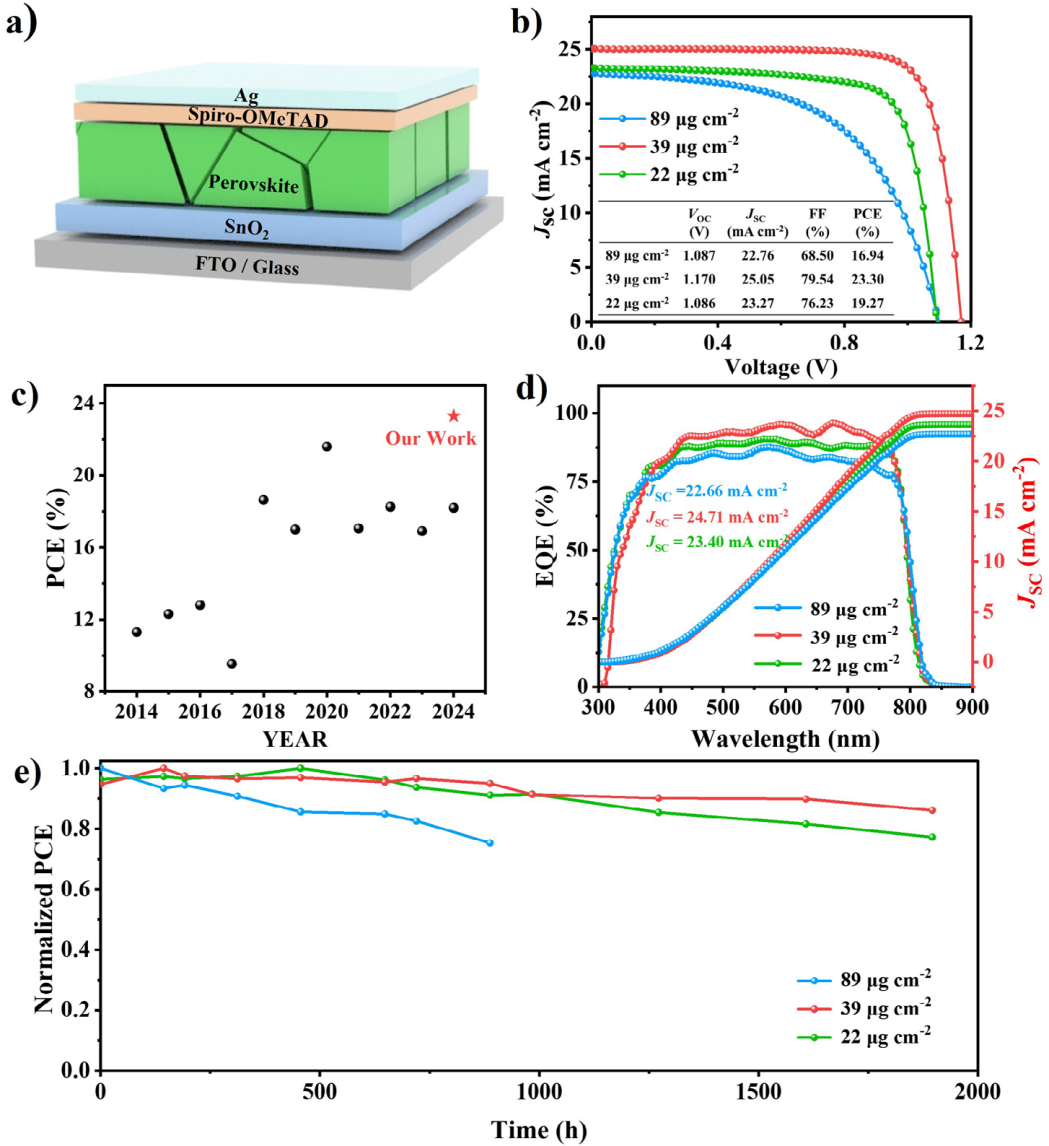
a) Schematic illustration of the device structure. b) Reverse scan *J*–*V* curves of the champion devices. c) Summary of the PCE evolution of devices fabricated via the inkjet printing method over time.^[^ ^27^, ^28^, ^32^, ^33^, ^36^, ^38^, ^66^, ^67^, ^68^, ^69^, ^70^ ^]^ d) IPCE spectra of the devices. e) Moisture stability test results under ambient air condition with RH of 20% at 25 °C.

To evaluate the performance and reproducibility of the devices at different organic salt deposition surface densities, PSCs with the structure of FTO/SnO_2_/Perovskite/Spiro‐OMeTAD/Ag were fabricated, as depicted in **Figure** **4**a. The statistics data for *J*_SC_, *V*_OC_, fill factor (FF), and PCE of the devices are presented in Figure S12 (Supporting Information). The devices fabricated with 89 µg cm^−2^ organic salts showed the lowest *J*_SC_, *V*_OC_, and FF values, which can be attributed to large pinholes at the perovskite grain boundaries, along with cracks through the perovskite film and at the perovskite/ETL interface, leading to inefficient charge carrier transport and increased film defects. In contrast, the devices prepared with 22 µg cm^−2^ organic salt showed improved *J*_SC_, *V*_OC_, and FF values, but these values did not reach their optimum due to excess PbI_2_, insufficient perovskite content and crystallization, and thinner films at lower deposition surface densities. At the optimal organic salt deposition surface density of 39 µg cm^−2^, a significant improvement in *V*_OC_ was observed, corresponding with reduced non‐radiative recombination as previously discussed. The enhanced *J*_SC_ in these devices indicated that a high‐quality perovskite film with optimal thickness and efficient charge separation and transport was achieved. Additionally, the dramatic improvement in FF further demonstrated the reduction of film defects and improved energy level alignment at the perovskite/HTL interface under optimal deposition conditions. Figure 4b presents the reverse scan *J*–*V* curves of the champion devices, along with their corresponding photovoltaic parameters. Devices prepared with 89 µg cm^−2^ organic salts showed the lowest PCE of 16.94%, with low *J*_SC_ (22.76 mA cm^−2^), *V*_OC_ (1.087 V), and FF (68.50%) values, owing to the low quality of the perovskite film. The devices fabricated with 22 µg cm^−2^ exhibited an improved PCE of 19.27%, with low *V*_OC_ (1.086 V), slightly improved *J*_SC_ (23.27 mA cm^−2^), and FF (76.23%) values. The slightly improved *J*_SC_ and FF values were attributed to reduced film defects and improved energy level alignment with the HTL. However, a relatively thin perovskite film with excess PbI_2_ resulted in a low *V*_OC_ value. The 39 µg cm^−2^ organic salt devices, benefiting from enhanced *J*_SC_ (25.05 mA cm^−2^), *V*_OC_ (1.170 V), and FF (79.54%), achieved the highest PCE of 23.30%, marking the best performance among inkjet‐printed devices (Figure 4c; Table S8, Supporting Information). The incident photon‐to‐current conversion efficiency (IPCE) spectra, shown in Figure 4d, yielded integrated current densities of 23.40, 24.71, and 22.66 mA cm^−2^ for the 22, 39, and 89 µg cm^−2^ devices, respectively, which corresponded well with the *J*_SC_ results from the *J*–*V* curves. Figure S13 (Supporting Information) presents the forward and reverse scans of the *J‐*‐*V* characterizations, with the corresponding device parameters listed in Table S7 (Supporting Information). The hysteresis values were 2.03%, 0.88%, and 5.94% for devices prepared with 22, 39, and 89 µg cm^−2^ of organic salt, respectively. The notably reduced hysteresis at the optimal deposition density of 39 µg cm^−2^ indicated the formation of a high‐quality perovskite film with fewer defects and enhanced charge carrier transport. These results confirm that optimal organic salt deposition plays a crucial role in minimizing hysteresis and improving device performance. To evaluate the effectiveness of our quantitative organic salt deposition strategy, we compared the statistics of the perovskite solar cells fabricated by inkjet‐printed organic salt at the optimum deposition surface density of 39 µg cm^−2^ with the cells prepared by the spin coating method. As shown in Figure S14 (Supporting Information), the inkjet‐printed PSCs exhibited slightly higher average *V*_OC_ and *J*_SC_, and thus a slightly higher average PCE of 22.24% than those prepared by the spin‐coating method (21.78%). This further proves the robustness of our quantitative organic salt deposition strategy. To assess long‐term stability, unencapsulated devices were tested under ambient conditions (20% relative humidity) for over 2000 h (Figure 4e). Devices prepared with 22 and 39 µg cm^−2^ organic salts retained 89% and 77% of their initial PCE, respectively, while the PCE of the 89 µg cm^−2^ devices dropped to 62% after 1000 h. These results demonstrated that perovskite films prepared at the optimal deposition surface density of 39 µg cm^−2^ significantly enhanced the long‐term stability of PSCs.

## 3 Conclusion

In summary, we highlight the critical role of precise organic salt deposition in optimizing perovskite film quality and enhancing the efficiency and stability of PSCs in this study. By developing a quantitative deposition approach via drop‐on‐demand inkjet printing, we demonstrated that controlling the organic salt surface density is essential for achieving a balanced perovskite crystallization process, minimizing defects, and improving charge carrier dynamics. Specifically, devices prepared with an optimal organic salt deposition surface density of 39 µg cm^−2^ achieved a champion PCE of 23.3%, the highest reported for inkjet‐printed PSCs. Moreover, the devices exhibited excellent long‐term environmental stability, retaining over 89% of their initial efficiency after 2000 h of storage in ambient air under 20% relative humidity. These findings not only deepen the understanding of film formation mechanisms in the two‐step fabrication method but also offer a scalable and reproducible strategy for enhancing PSC performance. Furthermore, this work has broader implications for enabling large‐scale, high‐throughput manufacturing of perovskite photovoltaics. The precise control of the deposition process through inkjet printing for high‐quality perovskite films paves the way for roll‐to‐roll fabrication, improving device uniformity while minimizing material waste, and ultimately accelerating the commercial viability of PSCs.

## Conflict of Interest

The authors declare no conflict of interest.

## Supporting information



Supporting Information

Supplemental Table 1

## Data Availability

The data that support the findings of this study are available in the supplementary material of this article.
