# Supplementary material for: Precise Control of Lead Halide and Ammonium Salt Stoichiometric Ratios for Efficient Perovskite Solar Cells
Source: Adv Sci (Weinh). 2025 Mar 20;12(18):2416634. doi: 10.1002/advs.202416634 (PMC12079538; doi:10.1002/advs.202416634)
Supplement: Supplementary file 1 — Supporting Information [file ADVS-12-2416634-s001.docx]

**Precise Control of Lead Halide and Ammonium Salt Stoichiometric Ratios for Efficient Perovskite Solar Cells**

*Hengyi Jiang^#^, Rui Yang^#^, Ziqi Zhu, Chao Sun, Yongbin Jin, Lingfang Zheng, Lina Shen, Chengbo Tian, Liqiang Xie, Jinxin Yang* and Zhanhua Wei**

Xiamen Key Laboratory of Optoelectronic Materials and Advanced Manufacturing, Institute of Luminescent Materials and Information Displays, College of Materials Science and Engineering, Huaqiao University, Xiamen 361021, China

E-mail: jinxinyang@hqu.edu.cn, weizhanhua@hqu.edu.cn

Supporting Information

Materials: Materials employed in the experiments include tin oxide colloid precursor (SnO_2_, 5% in H_2_O colloidal dispersion, J&K Scientific), cesium iodide (CsI, 99.9%, Sigma-Aldrich), lead iodide (PbI_2_, 99.99%, Sigma-Aldrich), guanidine hydroiodide (GAI, >97.0%, TCI), methylammonium iodide (MAI, Greatcell Solar), methylammonium bromide (MABr, Greatcell Solar), methylammonium chloride (MACl, > 99.5%, p-OLED), formamidinium iodide (FAI, 99.99%, Greatcell Solar), 2,2’,7,7’-tetrakis(N,N-p-dimethoxyphenylamino)-9,9’- spirobifluorene (spiro-OMeTAD, 99.8%, Shenzhen Feiming Technology Corporation), bis (trifluoromethane) sulfonimide lithium salt (Li-TFSI, 99.95%, Sigma-Aldrich), 4-tert-butylpyridine (TBP, 98%, SigmaAldrich), acetonitrile (99.8%, Sigma-Aldrich), dimethyl sulfoxide (DMSO, 99.9%, SigmaAldrich), N,N-dimethylformamide (DMF, 99.8%, Sigma-Aldrich), isopropanol (IPA, 99.5%, Sigma-Aldrich), chlorobenzene (CB, 99.8%, Sigma-Aldrich) and 1-Butanol (n-BuOH, 99.8%, Sigma-Aldrich).

Device fabrication: FTO conductive glasses were sequentially cleaned by ultrasonic cleaning for 20 min in glass cleaner, deionized water, ethyl alcohol, and isopropyl alcohol, respectively. Before the deposition of SnO_2_ ETL, a 10-minute plasma treatment was applied to the FTO substrates to increase the hydrophilicity of the surface. Afterward, SnO_2_ was spin-coated onto the FTO substrate at 4000 rpm for 20 s, and the SnO_2_ film was then annealed in air at 150 °C for 15 min. For the PbI_2_ layer, the PbI_2_ precursor solution was spin-coated onto the ETL at 2000 rpm for 30 s and then annealed in the glove box at 70 °C for 1 min. The organic salt solution was inkjet-printed onto the PbI_2_ by the Prtronic MP1100 printer equipped with a Dimatix printhead, then the film was annealed for 15 min at 150 °C in ambient air. For HTL, the Spiro-OMeTAD solution was spin-coated onto the perovskite film at 3000 rpm for 30 s. Eventually, a 90 nm Ag electrode was thermally evaporated onto the HTL.

*Characterization and Data Analysis:* The surface tension of the ink was measured by a contact angle meter (JC2000D, Powereach, Shanghai, China) in the pendent mode. The viscosity of the ink was measured by a rotational rheometer (MCR92, Anton Paar, Austria). The surface and cross-sectional structures of the samples were obtained via field-emission SEM (JEOL JSM-7610F plus), operated at an electron beam voltage of 3 kV. XRD measurements were carried out on a SmartLab X-ray diffractometer (Rigaku, Japan). The atomic force microscope (AFM) measurements were carried out in air conditions (Bruker Multimode 8) using frequency-3 modulation AFM to probe the Surface topography. UV-vis absorption spectra were collected on an equipment supplied by Xipu Electronics in the glovebox. Time-resolved photoluminescence (TRPL) was measured by FLS1000 (Edinburgh Instruments, Ltd.) with a pulsed excitation at 375 nm. J–V curves were collected by a Keithley 2400 source meter and an AAA-grade solar simulator (Enli. Tec.). The light intensity was calibrated by an NREL calibrated silicon solar cell equipped with an infrared cutoff filter (KG-5). The tested devices were measured with a black metal mask (0.119 cm^-2^). The test range of reverse scanning was 1.25 V to 0 V with a step rate of 0.02 V s^-1^. The space-charge-limited-current (SCLC) data were collected by the Keithley 2400 in the dark. The electrochemical impedance spectra (EIS) were measured on the CHI660E potentiostat system with a bias voltage of 1 V. The PL mapping image was measured by a laser scanning confocal microscope (Leica TCS SP8) with a pulsed excitation at 488 nm. The Mott-Schottky analysis and the transient photovoltage measurements were acquired on the Paios (Fluxim AG CH-8400 Winterthur, Switzerland). The EQE_EL_ was measured by a source meter (Keithley 2450) in the N_2_-filled glovebox. Incident photon-current efficiency (IPCE) data were obtained from the Enli QE-R equipment. Ultraviolet photoelectron spectroscopy (UPS) spectra were collected by a Thermo Fisher Scientific ESCALAB 250XI at a bias of -5 V using a He-Iα (21.22 eV) UV light source. XPS was performed by the Thermo Fisher Scientific K-alpha+. The statistical data of the devices were collected from fifteen PSCs for each organic salt deposition surface density gradient. The data of the *J*_SC_, *V*_OC_, FF, and PCE was analyzed by the box chart. The lower and upper boundaries of the box represented the first quartile (Q1) and the third quartile (Q3) of the data, respectively. The two outer horizontal lines represented the maximum and minimum values of the data. The data was fitted by a normal distribution.


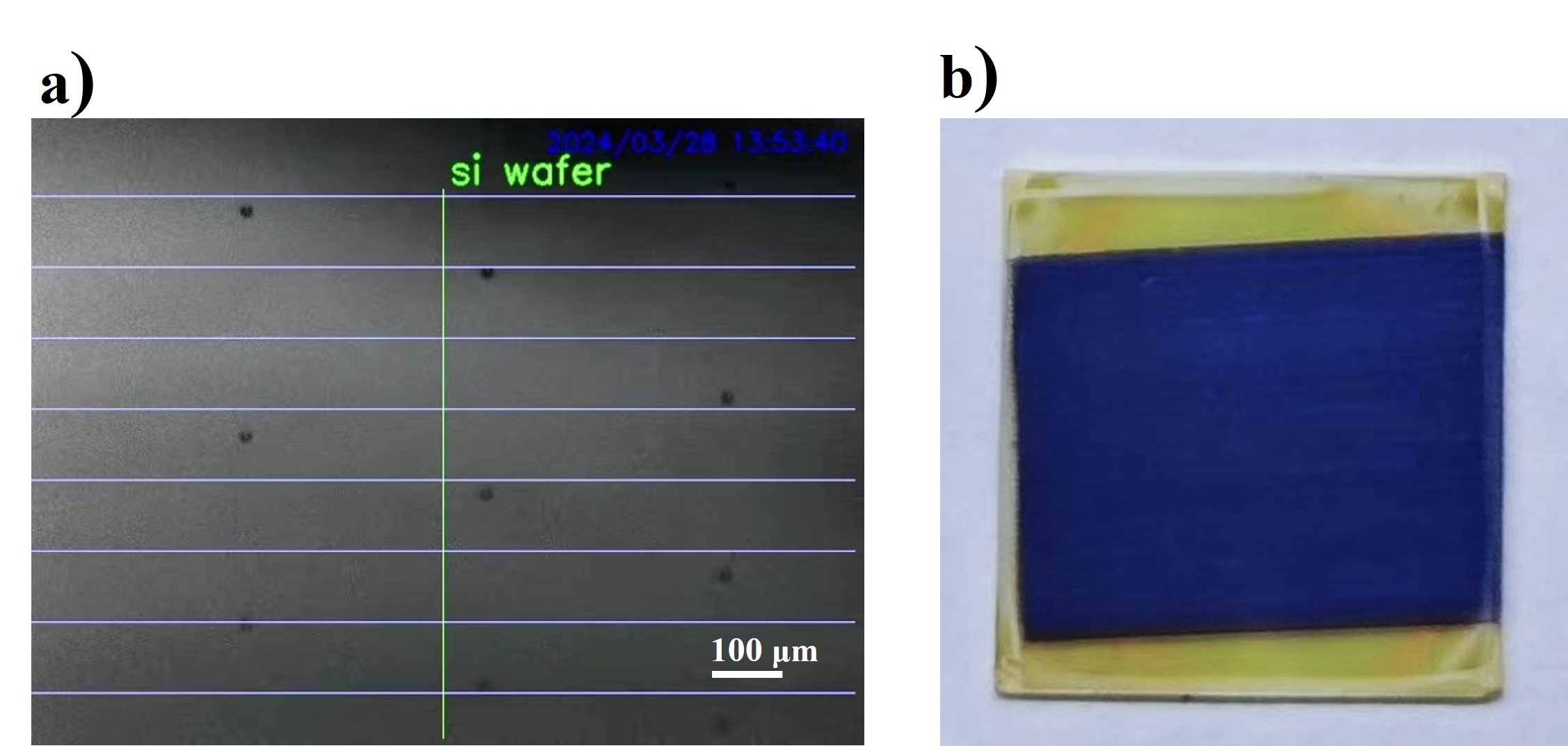


**Figure S1.** Photo of stable droplets of 59.3 mg mL^-1^ FAI in n-BuOH/IPA ejected from the nozzle during inkjet printing. b) Image of a perovskite film fabricated by inkjet printing. The uncovered PbI_2_ shows the advantage of patterning through inkjet printing, and also behaves as a watermark for inkjet-printed perovskite films.


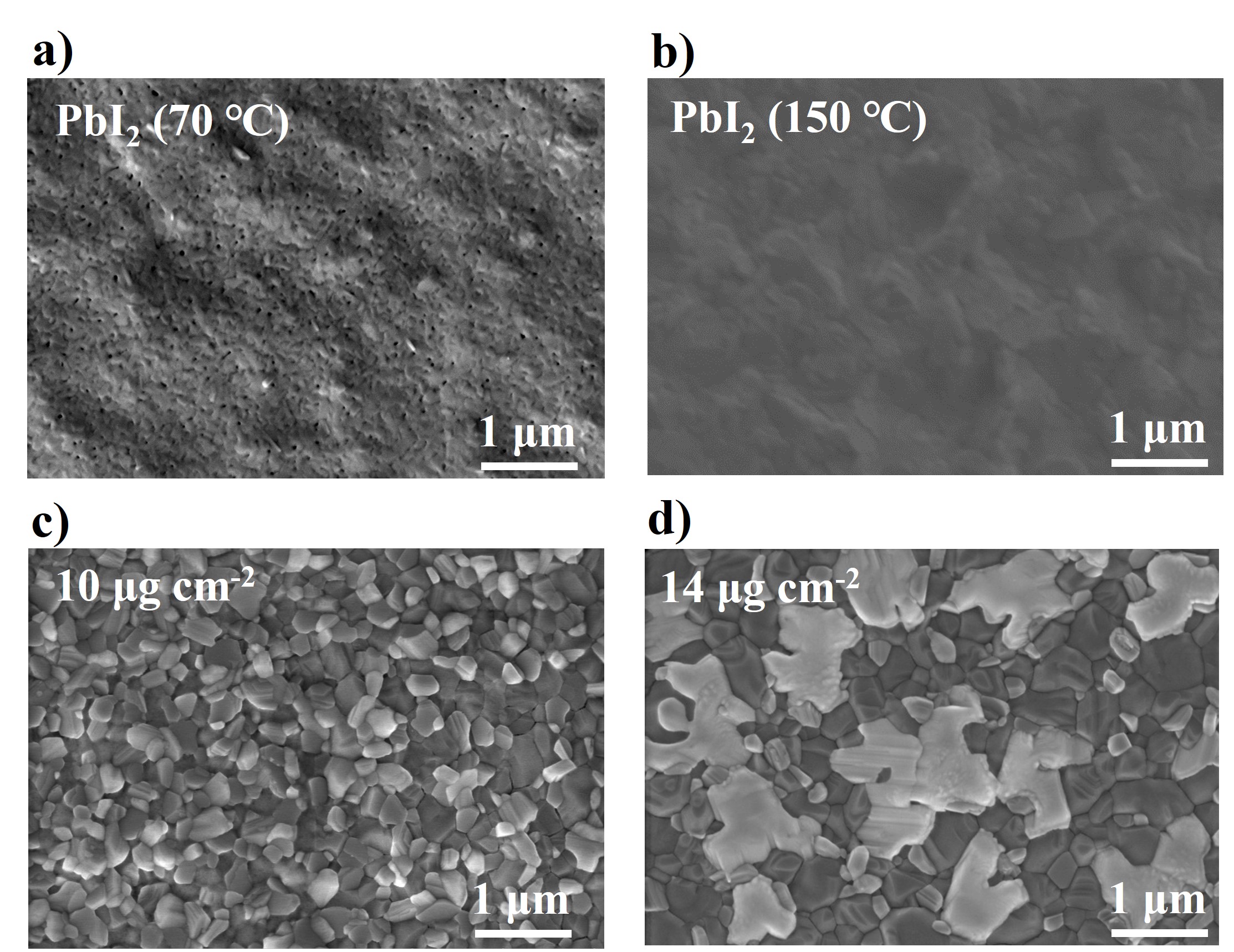


**Figure S2.** a-b) Top-view SEM images of the PbI_2_ films at different annealing temperature. c-d) Top-view SEM images of the perovskite films.


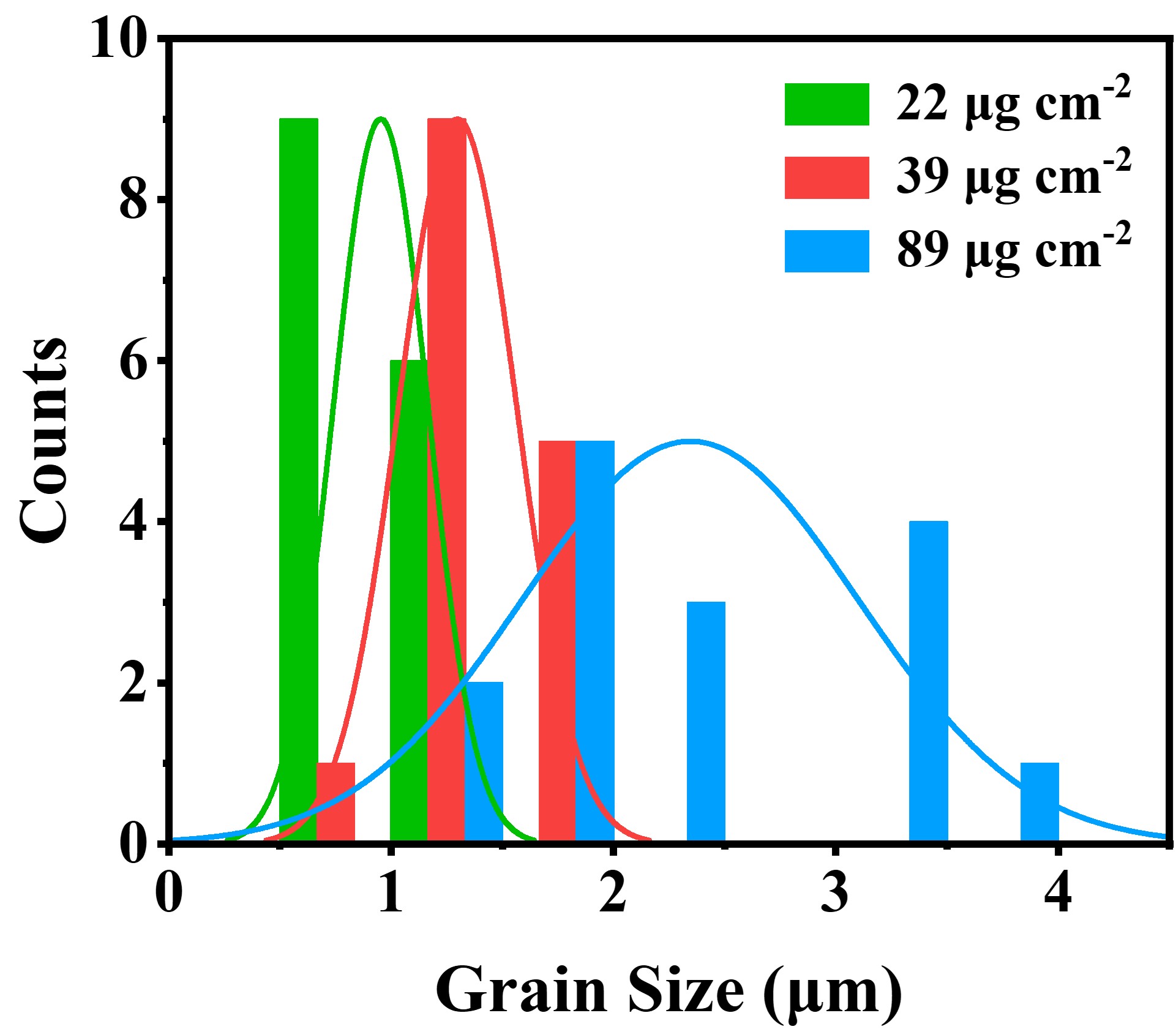


**Figure S3.** Perovskite grain size distribution at different organic salt deposition surface densities.


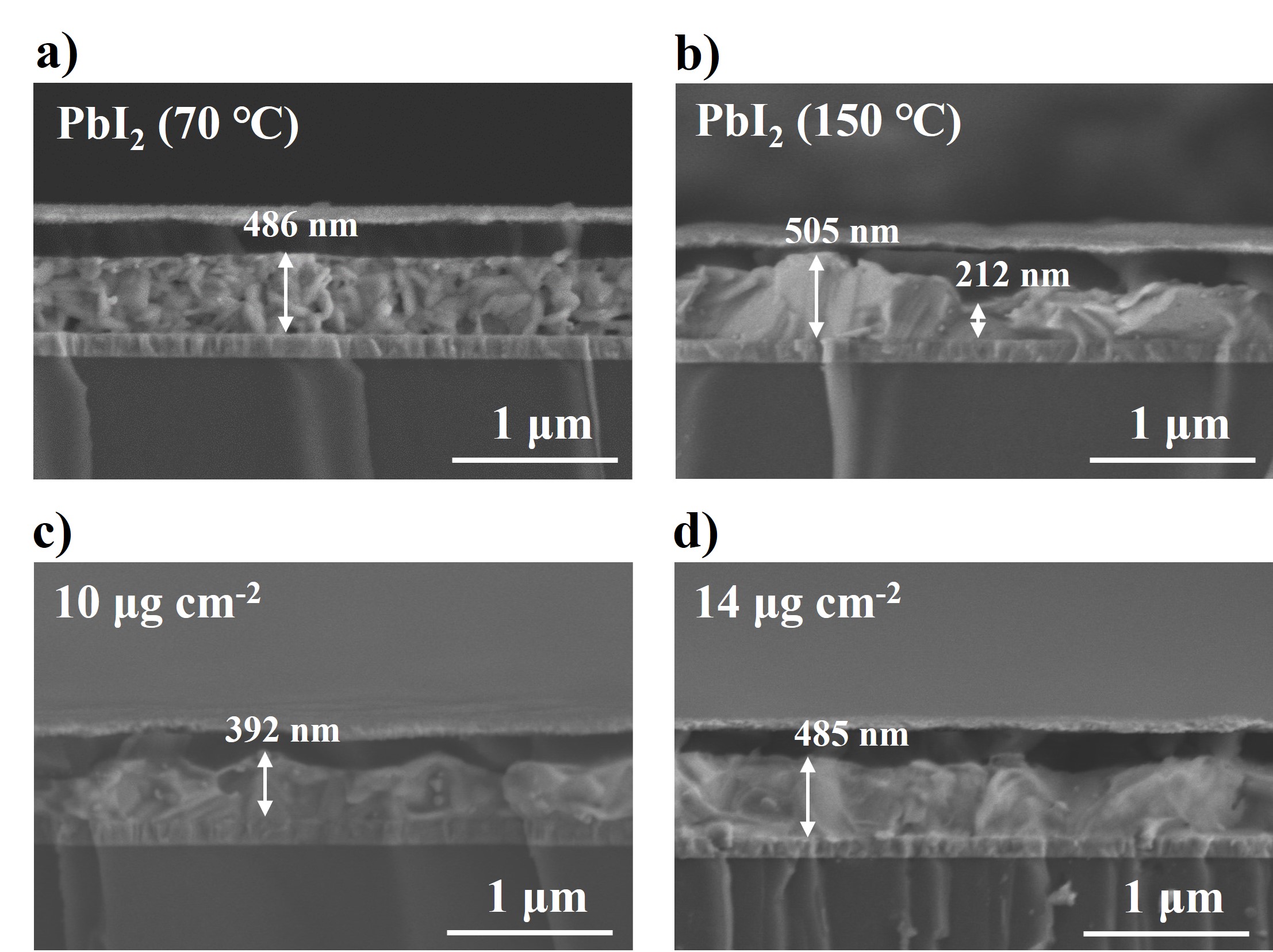


**Figure S4.** a-b) Cross-sectional SEM images of the PbI_2_ films. c-d) Cross-sectional SEM images of the perovskite films.

**
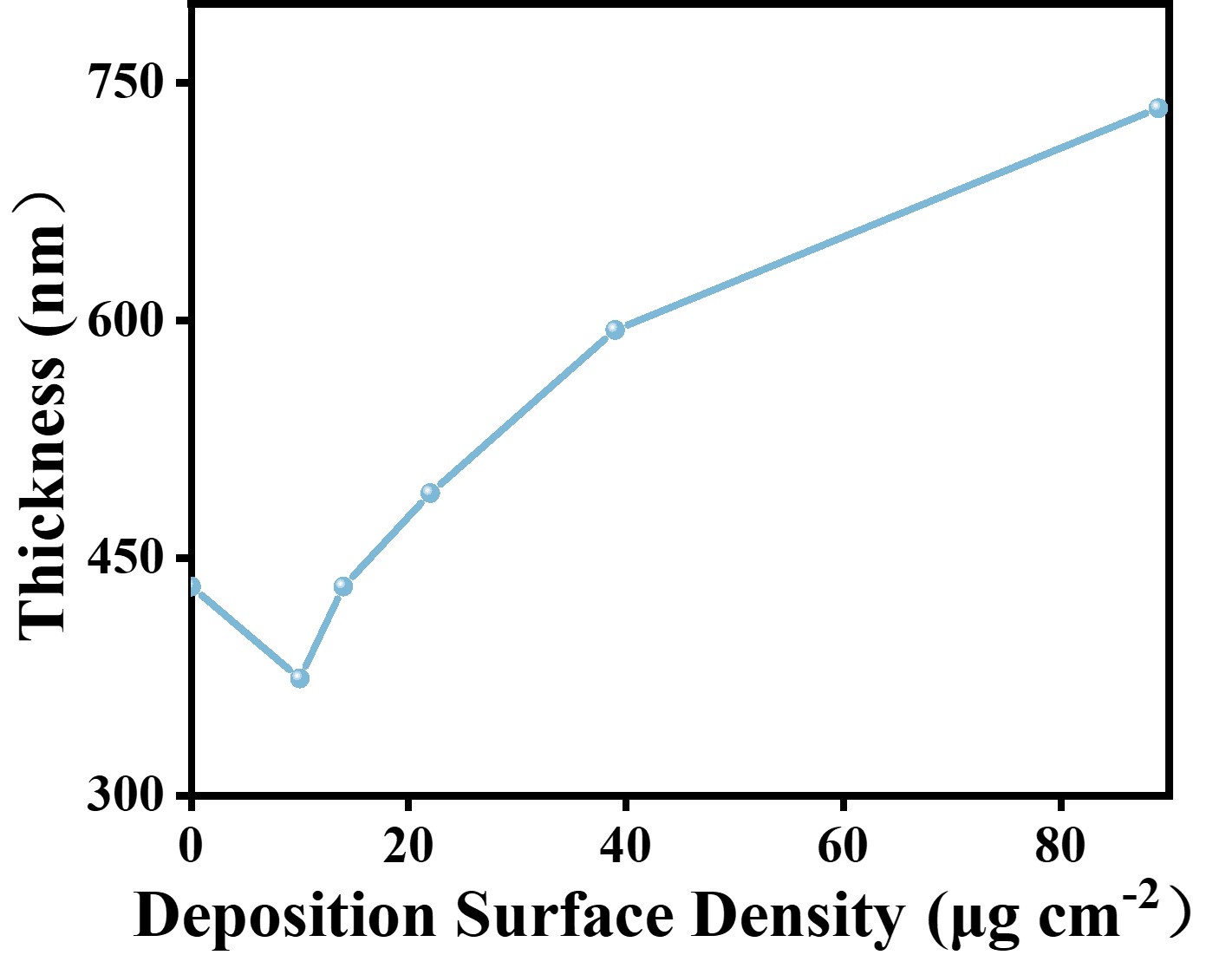
**

**Figure S5.** Relationship between perovskite film thickness and surface deposition density of the organic ammonium salt.

**
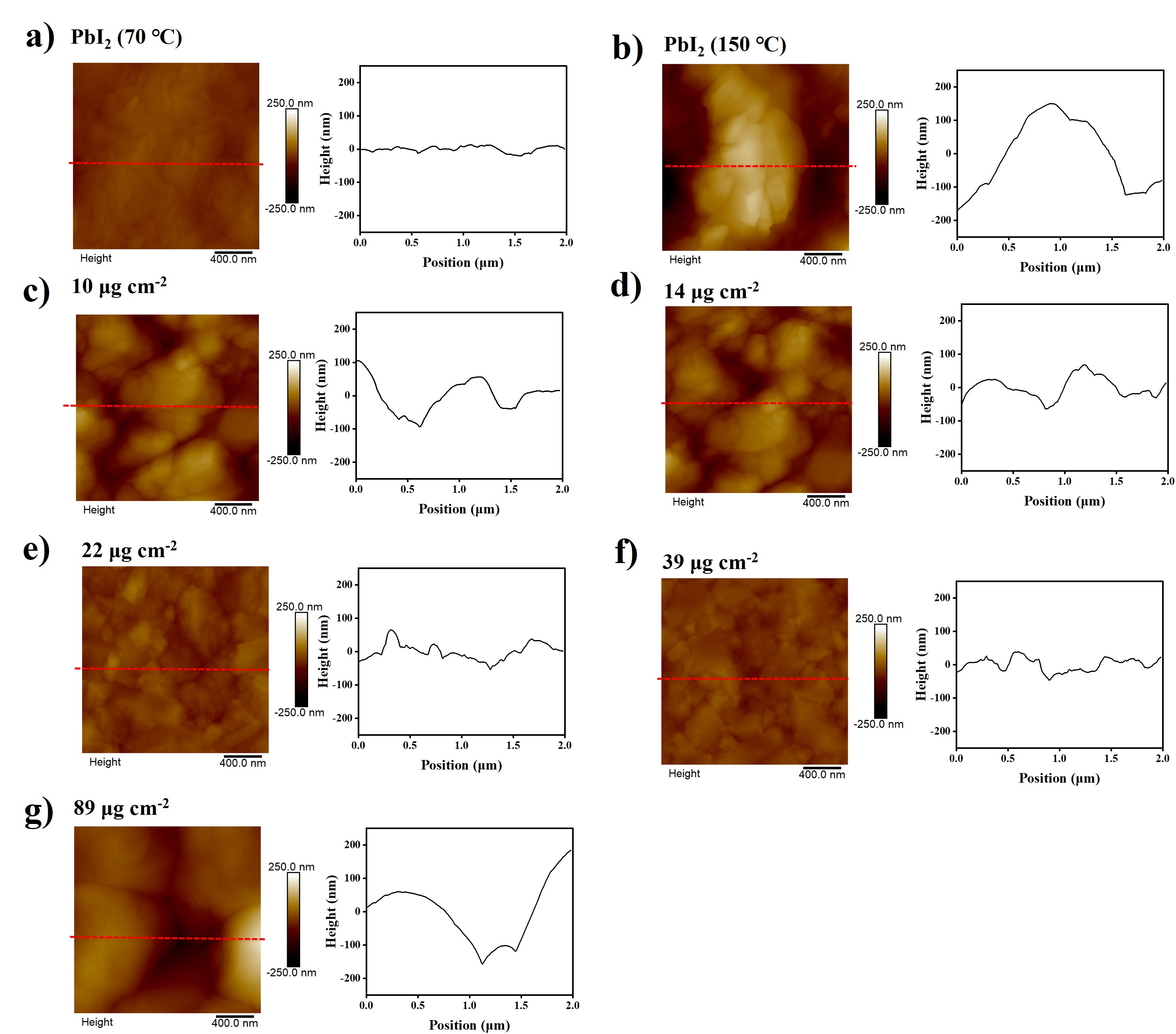
**

**Figure S6.** a-b) AFM images of the PbI_2_ films. c-g) AFM images of the perovskite films.

**
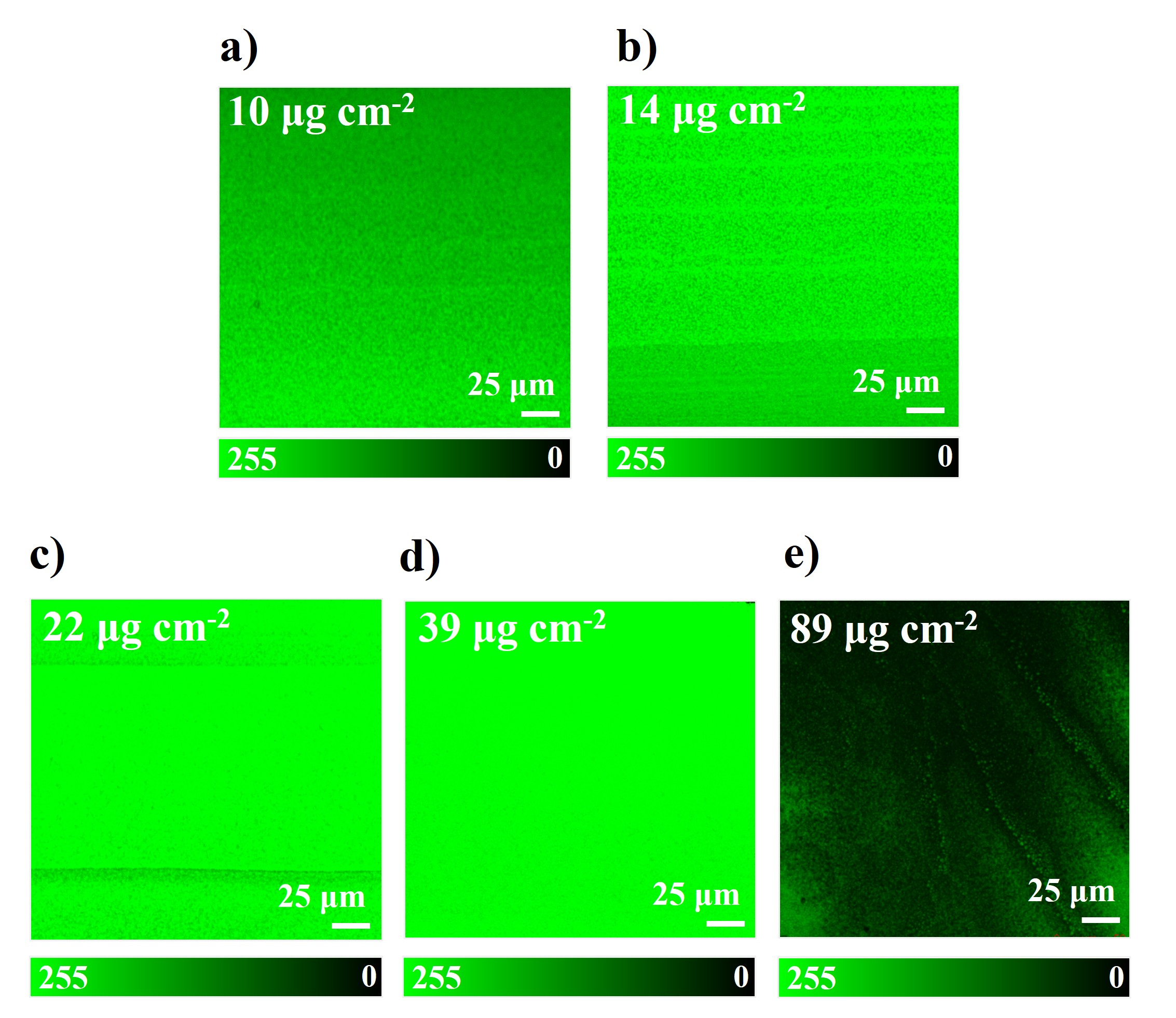
**

**Figure S7.** The PL mapping of the perovskite films at different organic salt deposition surface densities.

**
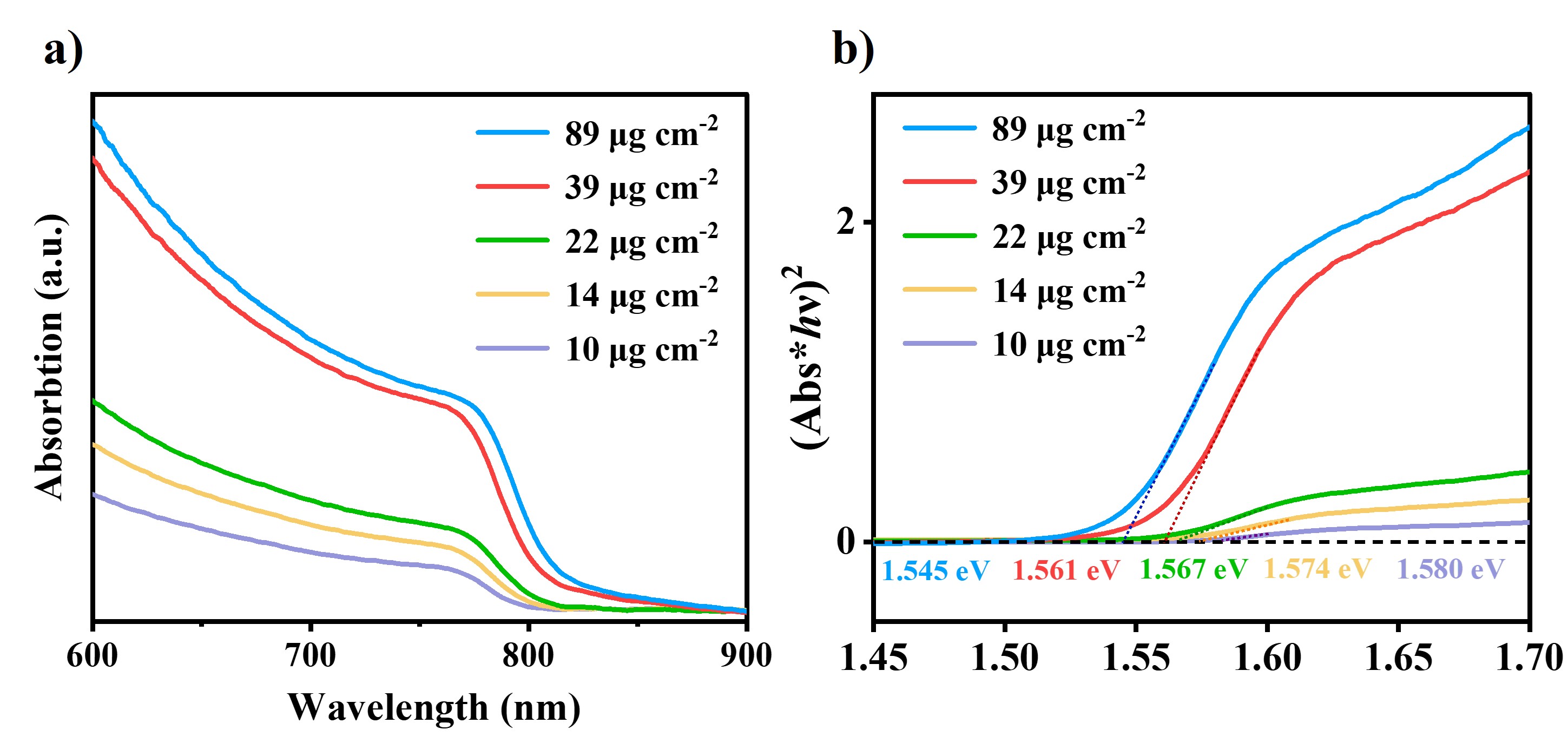
**

**Figure S8.** a) UV-vis spectra for the perovskite films at different organic salt deposition surface densities. b) Tauc plots from the corresponding UV-vis absorption spectra.

**
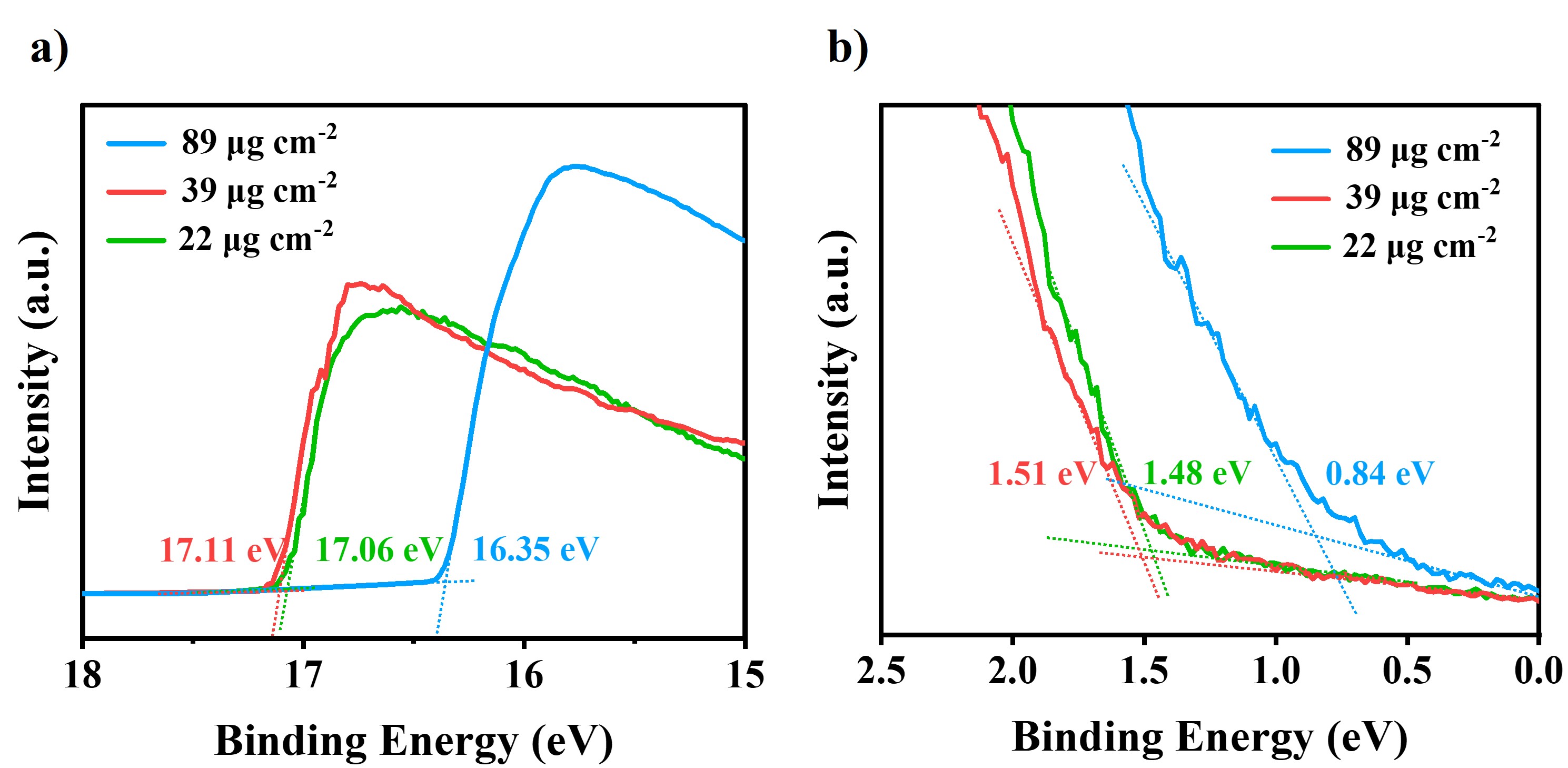
**

**Figure S9.** UPS spectra for the top surface of the perovskite films at different organic salt deposition surface densities.


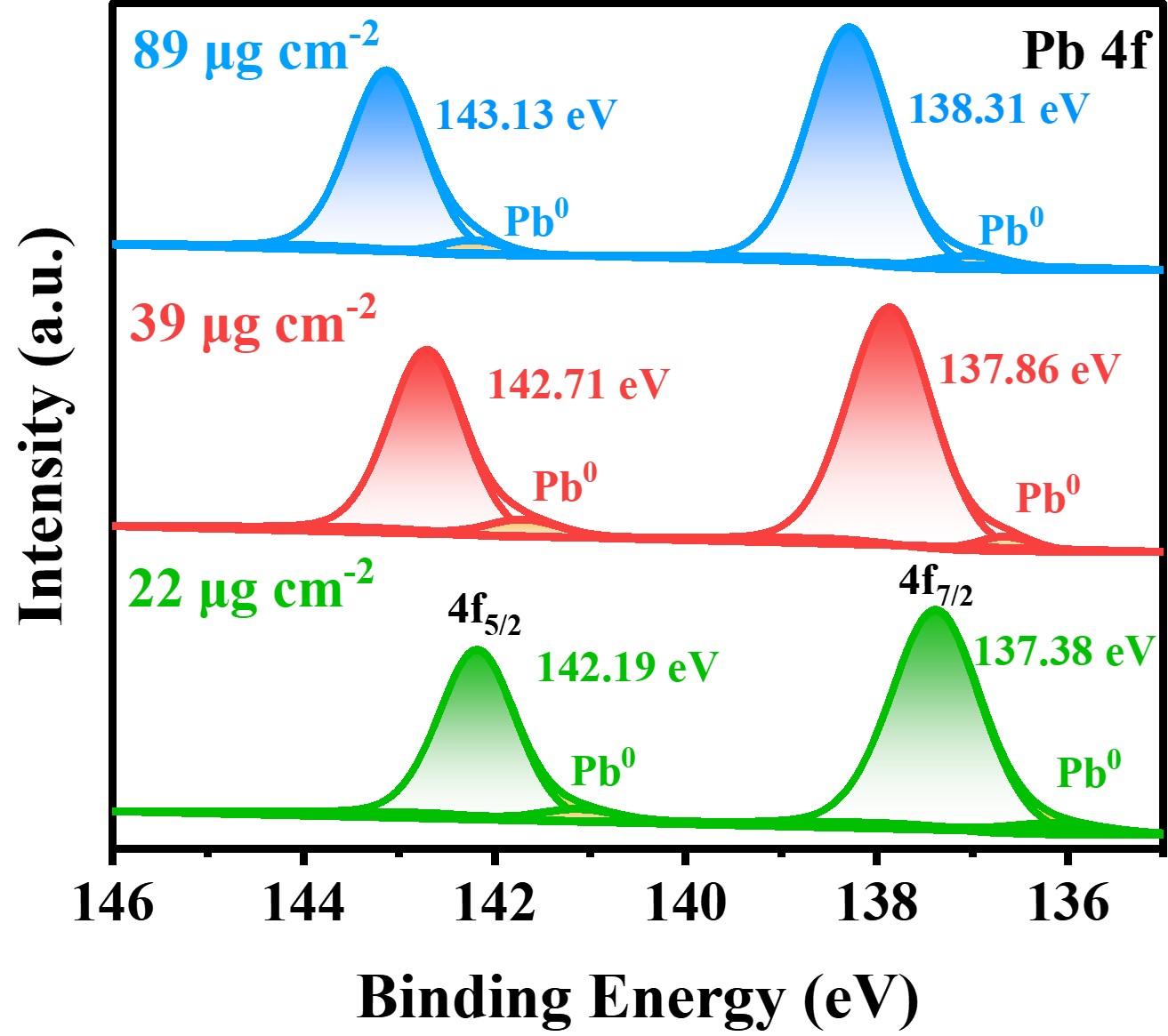


**Figure S10.** XPS spectra of Pb 4f orbital for perovskite films at different organic salt deposition surface densities.


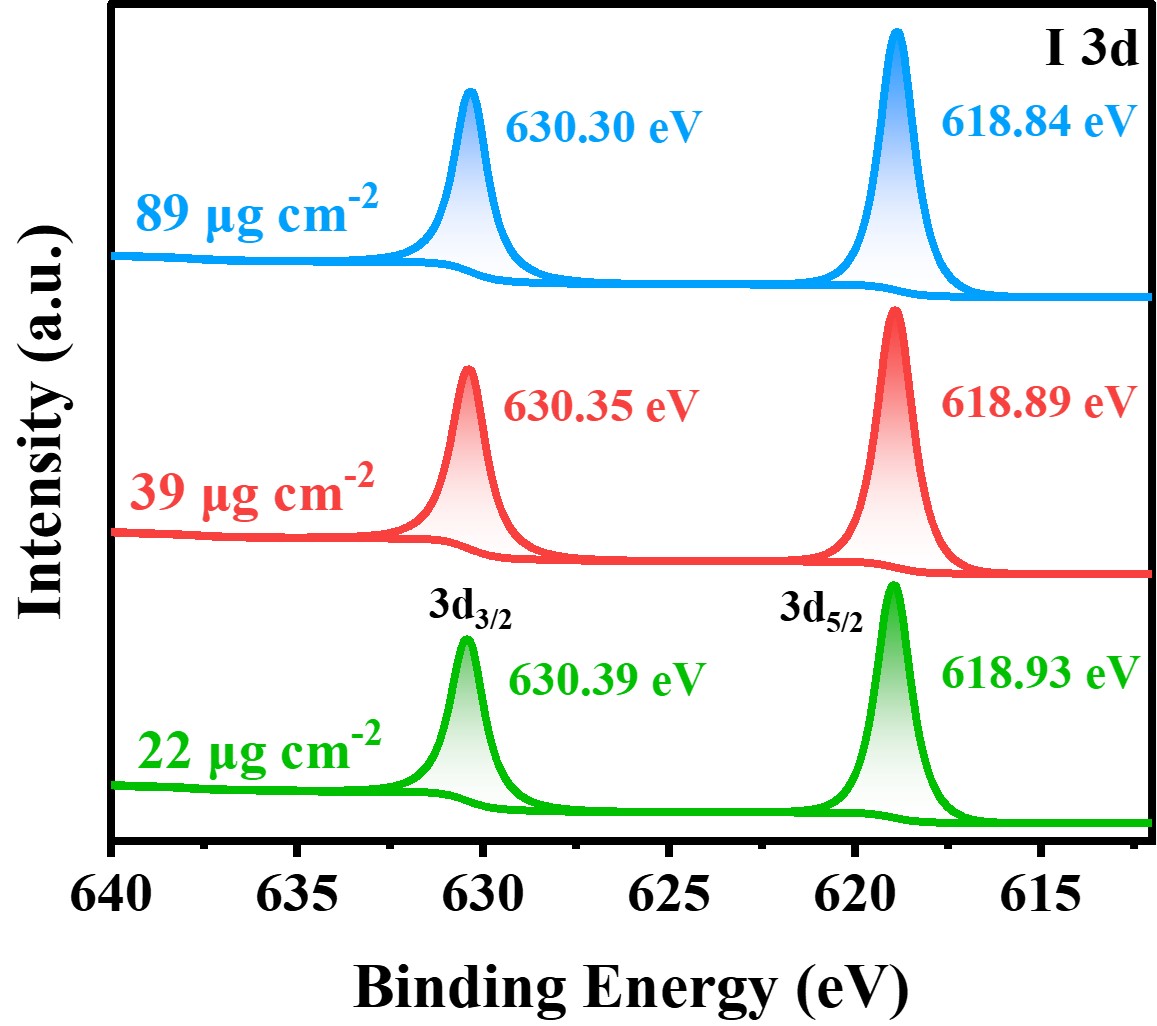


**Figure S11.** XPS spectra of I 3d orbital for perovskite films at different organic salt deposition surface densities.

**
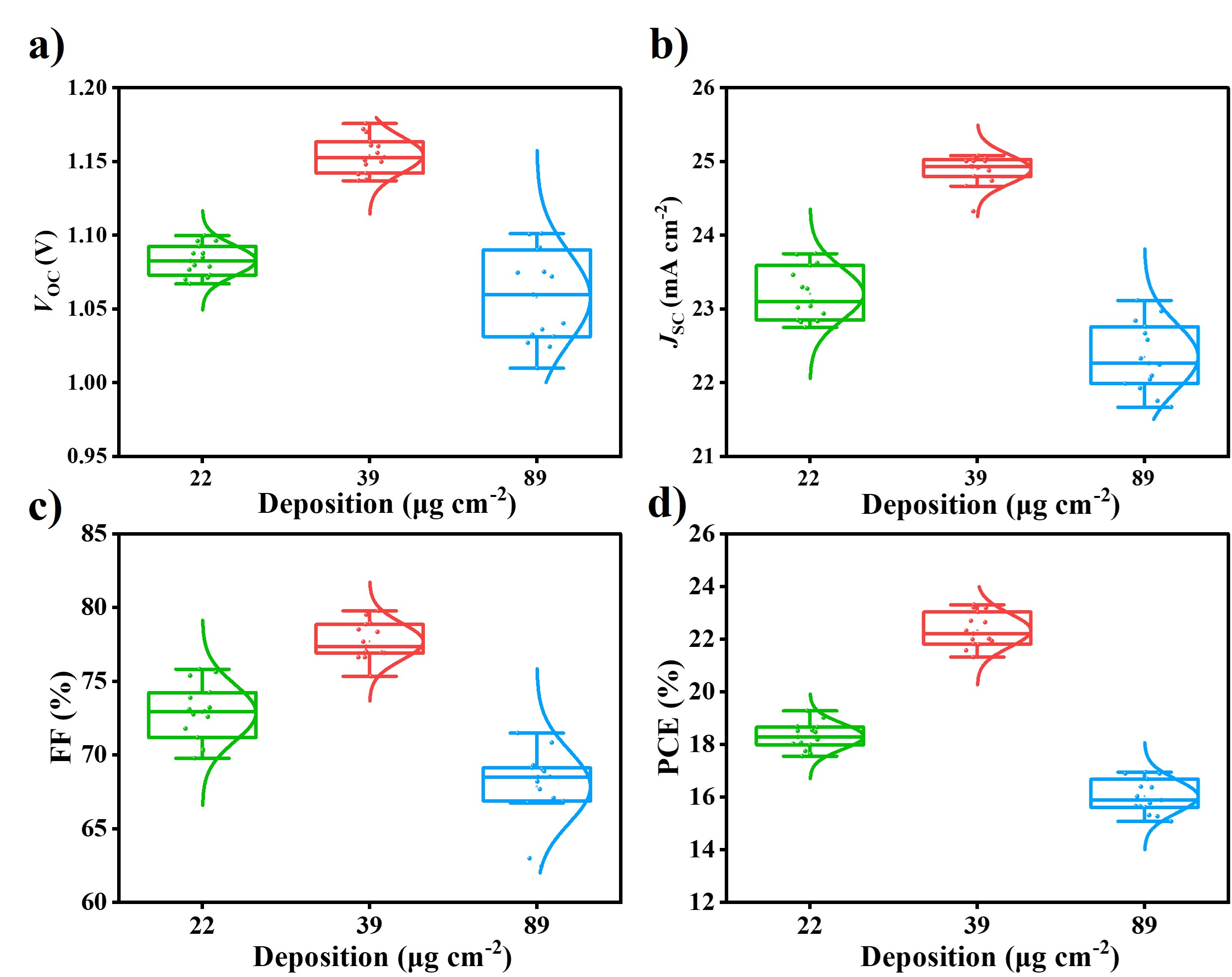
**

**Figure S12.** Statistics of the devices at different organic salt deposition surface densities: a) *V*_OC_, b) *J*_SC_, c) FF, and d) PCE.


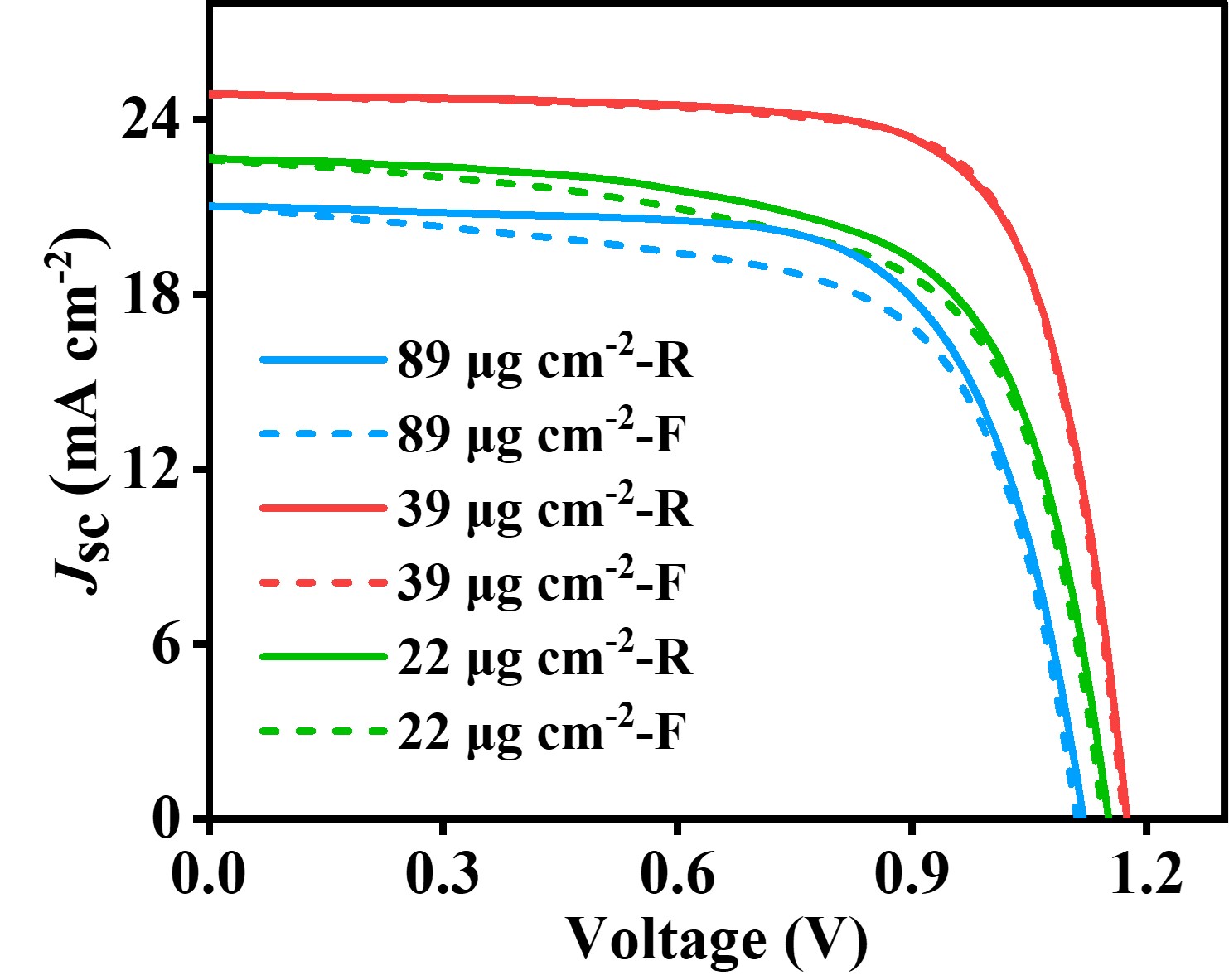


**Figure S13.** *J–V* curves measured under forward and reverse scans of the devices at different organic salt deposition surface densities.


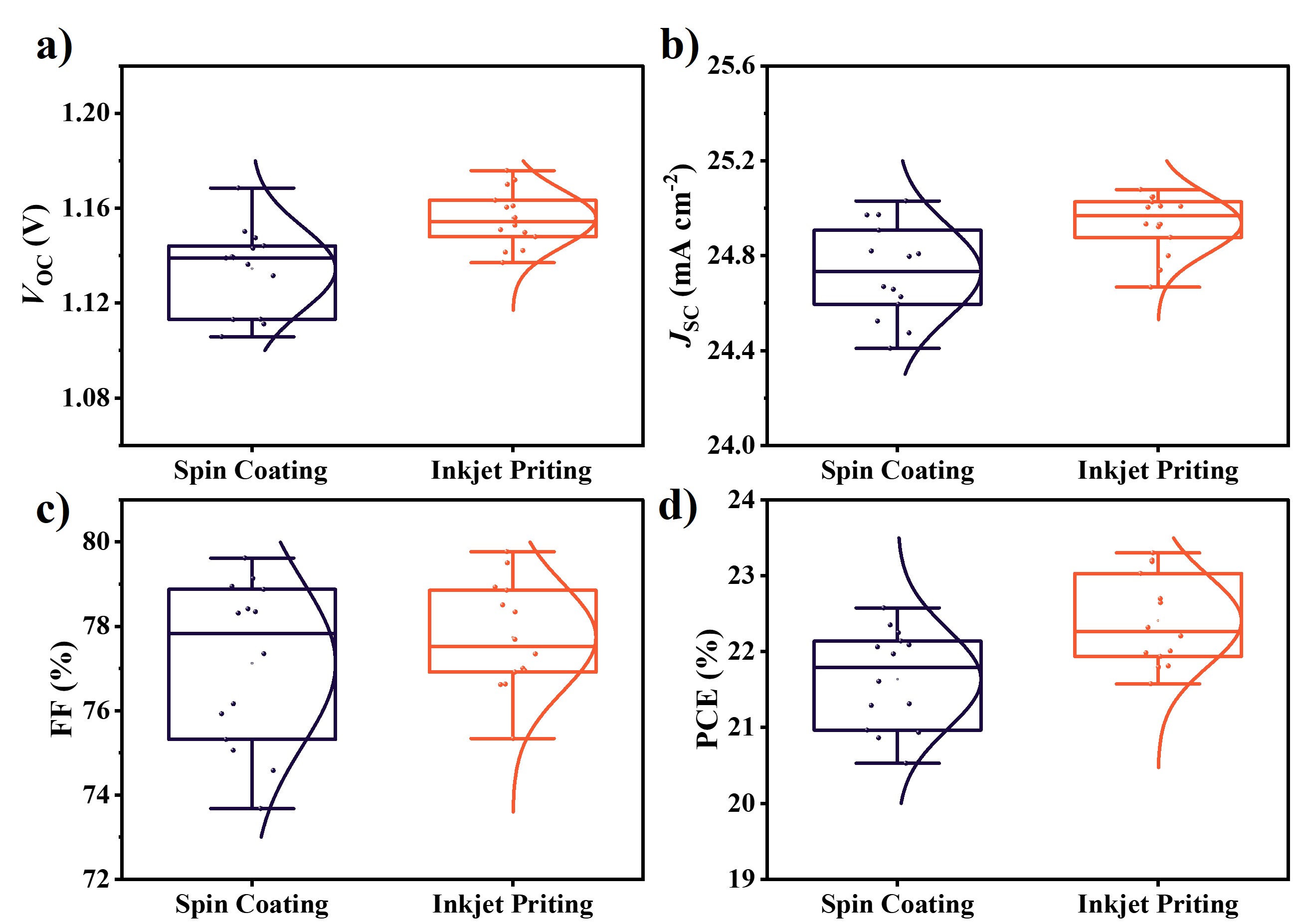


**Figure S14.** Statistics of the devices prepared by inkjet printing and spin coating: a) *V*_OC_, b) *J*_SC_, c) FF, and d) PCE.

**Table S1.** The rheological properties and *Z* number of the organic salt ink.

|  | ρ  (kg m^-3^) | η  (mPa·s) | γ  (mN m^-1^) | a  (m) | Z |
| --- | --- | --- | --- | --- | --- |
| 59.3 mg mL^-1^ FAI (n-BuOH:IPA=5:5) | 855.60 | 2.55 | 26.00 | 1.45*10^-5^ | 7.06 |

**Table S2.** The relationship between ink droplet spacing, droplet counts, deposition surface density, and film thickness.

|  | Droplet Counts  (cm^-2^) | Single Droplet Volume  (pL) | Ink  Concentration  (mg mL^-1^) | Deposition Surface Density  (μg cm^-2^) | Thickness  (nm) |
| --- | --- | --- | --- | --- | --- |
| 10 μm | 1.0×10^6^ | 1.5 | 59.3 | 89 | 734 |
| 15 μm | 4.4 ×10^5^ | 1.5 | 59.3 | 39 | 594 |
| 20 μm | 2.5×10^5^ | 1.5 | 59.3 | 22 | 491 |
| 25 μm | 1.6×10^5^ | 1.5 | 59.3 | 14 | 485 |
| 30 μm | 1.1×10^5^ | 1.5 | 59.3 | 10 | 392 |
| PbI_2_ (70 ℃) | - | - | - | - | 486 |
| PbI_2_ (150 ℃) | - | - | - | - | 505 / 212 |

**Table S3.** Average roughness of the perovskite films.

| Deposition Surface Density (μg cm^-2^) | *R*_a_ (nm) |
| --- | --- |
| PbI_2_ (70 ℃) | 11.3 |
| PbI_2_ (150 ℃) | 70.3 |
| 10 | 36.7 |
| 14 | 31.8 |
| 22 | 14.8 |
| 39 | 12.8 |
| 89 | 40.2 |

**Table S4.** XDR peak intensity ratio between PbI_2_ and perovskite 𝛼-(001).

| Deposition Surface Density (μg cm^-2^) | Peak Intensity Ratio PbI_2_/ 𝛼-(001) |
| --- | --- |
| PbI_2_ (70 ℃) | - |
| PbI_2_ (150 ℃) | - |
| 10 | 13.80 |
| 14 | 10.50 |
| 22 | 4.11 |
| 39 | 0.11 |
| 89 | 0.08 |

**Table S5.** The fitted results from the time-resolved PL decay curves of the perovskite films.

| Deposition Surface Density  （μg cm^-2^） | *A*_1_ | τ_1_  (ns) | *A*_2_ | τ_2_  (ns) | τ_ave_  (ns) |
| --- | --- | --- | --- | --- | --- |
| 10 | 709.14 | 160.97 | 186.71 | 340.14 | 225 |
| 14 | 478.09 | 177.65 | 405.74 | 352.24 | 287 |
| 22 | 427.31 | 152.20 | 434.40 | 657.18 | 563 |
| 39 | 483.71 | 163.32 | 397.43 | 860.36 | 729 |
| 89 | 771.23 | 87.35 | 121.52 | 270.09 | 147 |

**Table S6.** The EQE and Δ*V_OC_*_,norad_ of the devices at different organic salt deposition surface densities under the *J*_SC_ at AM 1.5G.

| Deposition Surface Density  （μg cm^-2^） | *J*_SC_ of AM 1.5G  (mA cm^-2^) | EQE  (%) | Δ*V_OC_*_,norad_  (mV) |
| --- | --- | --- | --- |
| 22 | 22.5 | 2.97 | 91.04 |
| 39 | 24.8 | 7.58 | 66.79 |
| 89 | 18.8 | 0.97 | 120.2 |

·

**Table S7.** Photovoltaic parameters of the 89, 39, and 22 μg cm^-2^ organic salt deposited devices under forward and reverse scan directions.

| Deposition Surface Density  （μg cm^-2^） | *V*_OC_  (V) | *J*_SC_  (mA cm^-2^) | FF  (%) | PCE  (%) | HI  (%) |
| --- | --- | --- | --- | --- | --- |
| 22 - R | 1.142 | 23.40 | 65.10 | 17.39 | 2.03 |
| 22 - F | 1.124 | 23.41 | 64.75 | 17.04 |  |
| 39 - R | 1.175 | 24.88 | 73.43 | 21.47 | 0.88 |
| 39 - F | 1.171 | 24.86 | 74.36 | 21.66 |  |
| 89 - R | 1.119 | 21.03 | 68.71 | 16.17 | 5.94 |
| 89 - F | 1.111 | 21.05 | 65.04 | 15.21 |  |

**Table S8.** Summary of efficiency of devices prepared by inkjet printing as a function of year.

| Year | Efficiency | Ref. |
| --- | --- | --- |
| 2014 | 11.60 % | 32 |
| 2015 | 12.30 % | 66 |
| 2016 | 12.80 % | 33 |
| 2017 | 9.53 % | 67 |
| 2018 | 18.64 % | 38 |
| 2019 | 19.00 % | 28 |
| 2020 | 21.60 % | 27 |
| 2021 | 17.20 % | 36 |
| 2022 | 18.26 % | 68 |
| 2023 | 16.90 % | 69 |
| 2024 | 18.20 % | 70 |
| 2024 | 23.30 % | Our Work |
